# The Plastic Age: River Pollution in China from Crop Production and Urbanization

**DOI:** 10.1021/acs.est.3c03374

**Published:** 2023-08-01

**Authors:** Yanan Li, Qi Zhang, Jantiene Baartman, Jikke van Wijnen, Nicolas Beriot, Carolien Kroeze, Mengru Wang, Wen Xu, Lin Ma, Kai Wang, Fusuo Zhang, Maryna Strokal

**Affiliations:** † College of Resources and Environmental Sciences, National Academy of Agriculture Green Development, Key Laboratory of Plant-Soil Interactions of MOE, China Agricultural University, Beijing 100193, China; ‡ Water Systems and Global Change Group, Wageningen University & Research, Droevendaalsesteeg 4, Wageningen 6708 PB, The Netherlands; § Soil Physics and Land Management Group, Wageningen University & Research, Droevendaalsesteeg 3, Wageningen 6708 PB, The Netherlands; ∥ Department of Science, Faculty of Management, Science & Technology, Open University, Heerlen 1081 HV, The Netherlands; ⊥ Key Laboratory of Agricultural Water Resources, Center for Agricultural Resources Research, Institute of Genetics and Developmental Biology, Chinese Academy of Sciences, 286 Huaizhong Road, Shijiazhuang 050021, China; # Environmental Systems Analysis Group, Wageningen University & Research, Droevendaalsesteeg 4, Wageningen 6708 PB, The Netherlands

**Keywords:** MARINA-Plastics (China-1.0), macro- and microplastics, agricultural plastic films, sewage, mismanaged solid waste

## Abstract

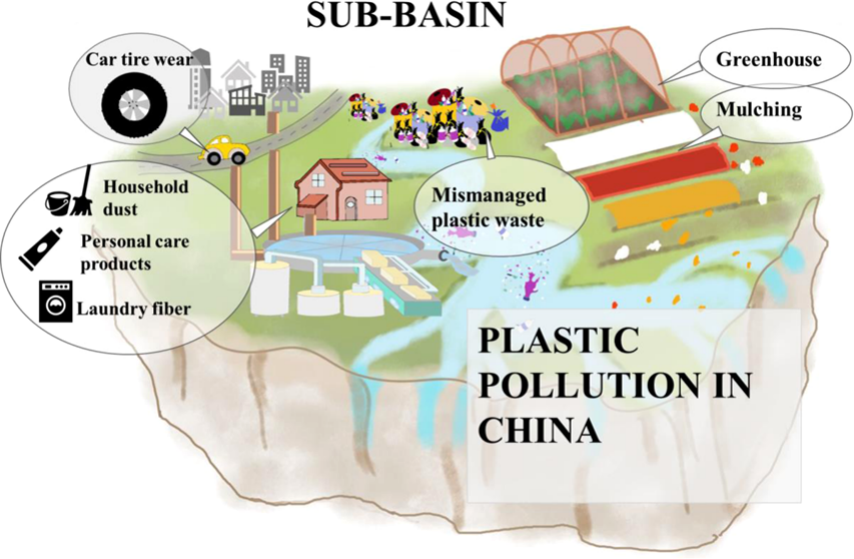

Many rivers are polluted with macro (>5 mm)- and microplastics (<5 mm). We assess plastic pollution in rivers from crop production and urbanization in 395 Chinese sub-basins. We develop and evaluate an integrated model (MARINA-Plastics model, China-1.0) that considers plastics in crop production (plastic films from mulching and greenhouses, diffuse sources), sewage systems (point sources), and mismanaged solid waste (diffuse source). Model results indicated that 716 kton of plastics entered Chinese rivers in 2015. Macroplastics in rivers account for 85% of the total amount of plastics (in mass). Around 71% of this total plastic is from about one-fifth of the basin area. These sub-basins are located in central and eastern China, and they are densely populated with intensive agricultural activities. Agricultural plastic films contribute 20% to plastics in Chinese rivers. Moreover, 65% of plastics are from mismanaged waste in urban and rural areas. Sewage is responsible for the majority of microplastics in rivers. Our study could support the design of plastic pollution control policies and thus contribute to green development in China and elsewhere.

## Abstract 2

Plastic pollution in rivers is modeled for nearly 400 Chinese sub-basins, and the importance of agriculture and urbanization is highlighted for pollution control.

## 1 Introduction

**Figure 1:**
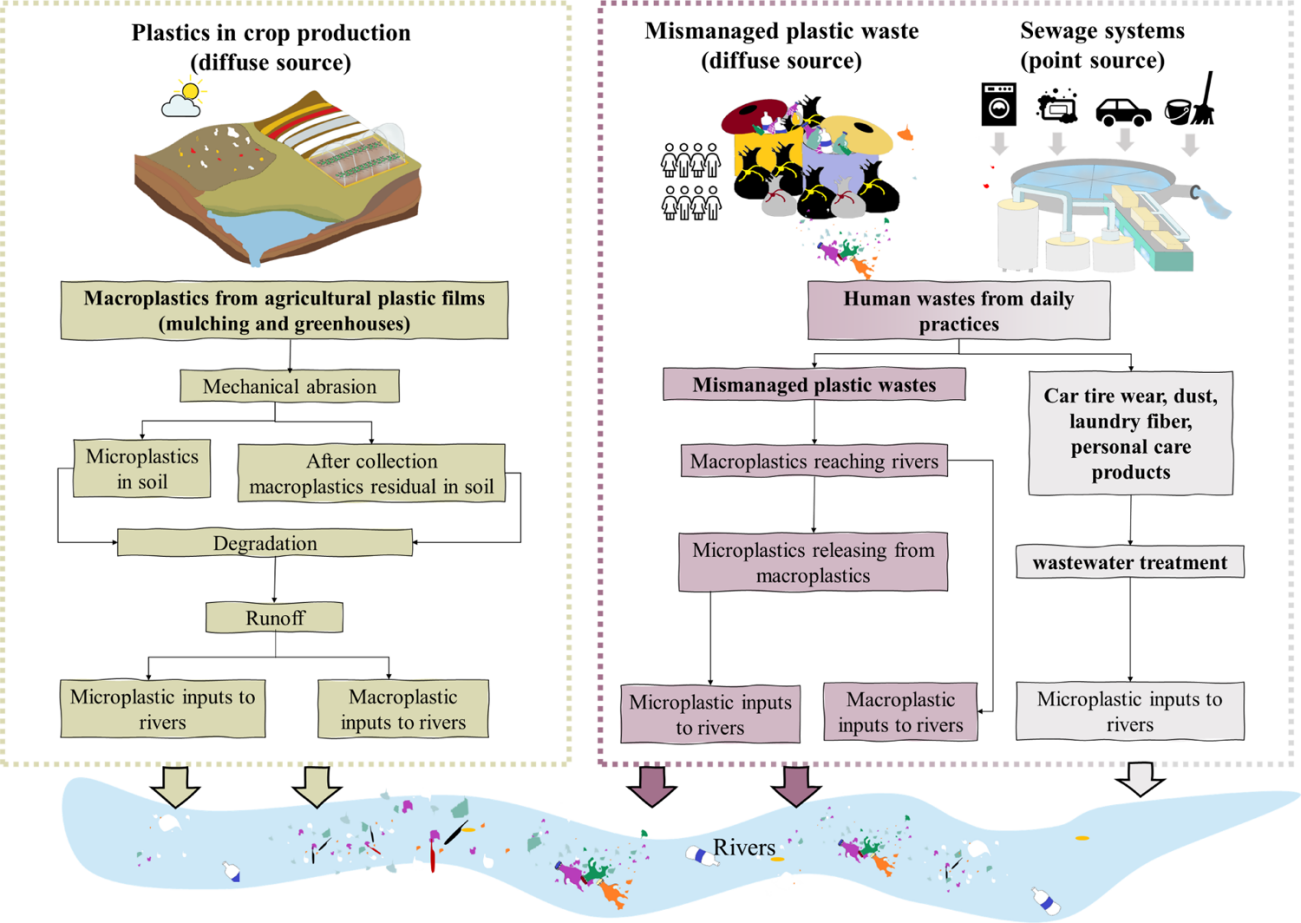
Main pathways of macro- and microplastic inputs to rivers from urbanization-related sources and crop production-related sources as implemented in the MARINA-Plastic model (China-1.0). Source: literature as reviewed in the Introduction and Materials and Methods sections.

One may define this century as the “Plastic Age” in which the use of plastic products has increased worldwide.^1^ A side effect of this is that plastics enter the environment and accumulate in soils,^2,3^ sediments,^4^ and water.^5−7^ Larger plastic debris (macroplastics >5 mm) can break down into microplastics (<5 mm) causing secondary pollution.^1,7,8^ Both macro- and microplastics with their additives pose a threat to society and nature such as accumulation in the food web of aquatic systems and damage to infrastructures.^9−13^ China is a country with increasing use of plastics because of urbanization and crop production.^14,15^ Urbanization can contribute to macroplastics in rivers from mismanaged solid waste^16^ and microplastics from sewage systems^17^ (Figure 1). Mismanaged solid waste is a diffuse source because of runoff from streets to nearby water systems.^18,19^ Sewage systems contain microplastics from car tire wear, personal care products, laundry fiber, and household dust.^17,18^ Sewage systems are point sources of microplastic pollution. Crop production contributes to plastics in rivers from the mulching of cropland and greenhouses (diffuse sources).^20^ Mulching is used to cover crops with plastics. Greenhouses are used to grow, for example, vegetables. These are often diffuse sources of plastic pollution because of runoff from cropland to rivers. Other plastic sources are architectural coatings,^21^ landfill waste, and manufactory waste.^22^

Potential plastic sources are identified; however, the relative contribution of each is not well studied. For instance, Wang et al.^21^ indicate that 54 and 29% of the total microplastics are from tire dust and synthetic fiber in China; however, the contributions of other sources are not clear. Furthermore, existing studies often focus on either macro- or microplastics,^16,17,23−26^ covering the world or specific basins,^8,17−19,25^ and are limited to either urban or agricultural sources. Modeling approaches to quantify plastic inputs to aquatic systems exist for macroplastics and microplastics,^18,19,26^ but those models are limited to the sources and scale (e.g., basin versus country) and they do not focus especially on China. Mulching and greenhouses in crop production are often ignored in those models. Recently, the first sub-basin scale model accounting for both macro- and microplastics has been developed (MARINA-Plastics Global-1.0 model).^27^ This model considers the following sources: mismanaged solid waste (diffuse source of macroplastics), sewage systems (point source of microplastics), and the fragmentation of macroplastics into microplastics (diffuse source of microplastics). However, they ignored agricultural plastic films from crop production as a source of macro- and microplastic in rivers.

According to the Chinese State Oceanic Administration, 81% of coastal waters experience plastic pollution in China.^10^ This situation could be worse in the future because of urbanization and intensive agriculture.^28,29^ Rivers are considered the dominant contributors to plastic pollution in the ocean.^26,28^ Currently, the Chinese government promulgates policies for plastic pollution reduction, but focuses more on plastic pollution management in urban areas, compared to agriculture (See Table S1).^30^ In 2012, around 13% of Chinese cropland was mulched with agricultural plastic films, which accounts for 60% of mulched cropland area globally.^31^ Thus, a comprehensive assessment of how crop production (mulching and greenhouses) affects river pollution with plastics is needed. This will form the basis for designing plastic reduction management strategies where both urbanization and crop production are considered. Such strategies will also contribute to green development in China, which aims at sustainable crop production and a clean environment.

In this study, we assess the implications of crop production and urbanization on river pollution with plastics in China. To this end, we develop and evaluate a new version of the MARINA-Plastics model that explicitly considers crop production and urbanization for the year 2015. Our study focuses on 395 river sub-basins in China and highlights macro- and microplastic pollution hotspots. Our results contribute to raising public awareness of plastic pollution and could inspire regional governments for formulating strategies to support future green development. The new model can serve as a basis for similar assessments in other regions of the world.

## 2 Materials and Methods

We developed a new version of the MARINA-Plastics model for China based on the existing models. Below, we first introduced the existing version MARINA-Plastics (Global-1.0). Next, we described our newly developed version MARINA-Plastics (China-1.0).

### 2.1 Existing MARINA-Plastics Model (Global-1.0)

The MARINA-Plastics model (Global-1.0) is a **M**odel to **A**ssess **R**iver **I**nputs of polluta**N**ts to se**A** for **Plastics** on a global scale. This model was developed based on existing sub-basin modeling approaches from Strokal et al.^17^ and plastic models from van Wijnen et al.^18^ and Siegfried et al.^32^ MARINA-Plastics (Global-1.0) can quantify annual river export of micro and macroplastics by sources from sub-basins to coastal waters of the world. This model can run for 10,226 river sub-basins globally. This model mainly focused on urbanization-related sources including mismanaged solid waste and sewage systems originating from the urban and rural populations. Mismanaged solid waste refers to plastic waste that stays on land without proper management and collection. Mismanaged solid waste is a diffuse source of macro- and microplastics in rivers. Sewage systems are point sources of microplastics in rivers. Sewage systems that discharge microplastics to rivers from laundry fibers, household dust, car tire wear, and personal care products.^17^ Agricultural sources are not considered in this existing MARINA-Plastics (Global-1.0) model.

### 2.2 New MARINA-Plastics Model (China-1.0)

We took the MARINA-Plastics model (Global-1.0) and developed it further for 395 Chinese sub-basins. The MARINA-Plastics model (China-1.0) is used to quantify the annual input of macro- and microplastics to rivers and sources in 395 sub-basins in China for the year 2015 (Figure 1). MARINA-Plastics (China-1.0) is a deterministic (not stochastic) and uncalibrated model. The model considers crop production-related sources and urbanization-related sources (Figure 1, Table S2). Crop production-related sources include agricultural plastic films, which are plastics from mulching and greenhouses. After agricultural plastic film application on the cropland, not all plastics are collected. A certain amount (macro- and microplastics) stays on the land and can enter rivers via a diffuse pathway (e.g., surface runoff). These are diffuse sources of water pollution. Urbanization-related sources refer to sewage systems and mismanaged solid waste resulting from urban and rural populations. Sewage systems are the pipes discharging effluents (containing microplastics) to rivers after treatment. We consider that inputs of microplastics to rivers resulting from sewage systems are point sources of water pollution. However, not all microplastics from car tire wear reach sewage systems. Some microplastics can stay on nearby land and reach rivers via runoff as a diffuse source. We do not consider this diffuse source in our model because of data availability. Mismanaged solid waste is not managed properly and contains plastics that can enter rivers in a diffuse matter (e.g., via surface runoff). This is a diffuse source of water pollution that we consider in our model.

Our new MARINA-Plastics (China-1.0) model differs from the original version in two main aspects. *First*, we developed a new modeling approach to calculate the annual input of macro- and microplastics to rivers from mulching and greenhouse plastics in crop production as inspired by existing studies.^33−35^*Second*, we updated the local information about macro- and microplastic inputs to rivers in 395 Chinese sub-basins for the year 2015. The data sources of our model inputs and data processing are presented in Supplementary Information Tables S3 and S4.

For plastics from crop production, we took a lumped approach to quantify the annual input of macro- and microplastics to rivers from agricultural plastic films in the MARINA-Plastics model (China-1.0). We accounted for the transformation, degradation, and transportation processes of macro- and microplastics from cropland to rivers (Figure 1). For example, we considered microplastic inputs to land from the mechanical abrasion of agricultural plastic films. After plastics are collected from land, their films are left partly in soils as macroplastic residues. Macro- and microplastic in soils can degrade and be transported to rivers via surface runoff. We also consider the impact of urbanization on water pollution. Urbanization includes the effects of human activities in urban and rural areas related to sewage connections of urban and rural populations and mismanaged solid waste that is produced from the total population. For plastics from urbanization-related sources, we followed the MARINA-Plastics model (Global-1.0) approach to quantify macro- and microplastic inputs to rivers. Below, we explained how we calculated the annual input of macro- and microplastics to rivers from crop production and urbanization-related sources.

### 2.3 Inputs of Macro- and Microplastics to Rivers from Crop Production

The total amount of plastics in rivers from crop production is the sum of macro- and microplastics from agricultural plastic films (1). The annual input of macro- and microplastics to rivers from agricultural plastic films is quantified in two steps. *First*, we quantified macro- and microplastic residues in soils from the application of agricultural plastic films (2 and 3). This was done as a function of the mechanical abrasion, solar radiation, and residue rates of macroplastics in soils. The mechanical abrasion depends on plastic materials. Polyethylene (PE) and poly(vinyl chloride) (PVC) are the dominant categories of agricultural plastic films in China.^34^ Here, we considered the mechanical abrasion for both PE and PVC based on the study of Ren et al.^33^ (Tables S3 and S4). Moreover, we distributed the residue rate for macroplastics in soils at the sub-basin scale based on the study of Zhang et al.^35^ The residue rate implies that macroplastics are left in the soil after agricultural plastic films are collected (Tables S3 and S4).

*Second*, we accounted for macro- and microplastic degradation in soils as a function of various processes and surface runoff (4 and 5). The degradation rate influences losses of macro- and microplastics during export from land to rivers. Physical (e.g., solar radiation-related plastic degradation), chemical (e.g., soil pH-related plastic degradation), and biological (e.g.,microorganisms-related degradation) processes influence the degradation rates of plastics in soils (Figure S1, Table S5). We estimated the degradation rate of plastics in soil based on the existing experimental studies (Table S5). Plastics can be decomposed into carbon dioxide, organic matter, and water. We assigned the plastic degradation rate of 1–5% in soils to sub-basins based on the results of physical, chemical, and biological processes (see Tables S3–S5, Figure S1). Annual input of macro- and microplastics to rivers is influenced by runoff and precipitation (4 and 5). In general, more runoff means more chance that plastics enter rivers compared to sub-basins with less runoff. Steep landscapes generally have a higher velocity of runoff. Our model used runoff for up-, middle-, and downstream sub-basins.

The equations of MARINA-Plastics (China-1.0) for calculating the annual input of macro- and microplastics from agricultural plastic films (crop production) are as follows
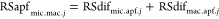
1
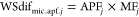
2
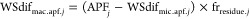
3
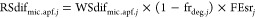
4
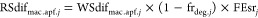
5where,

**RSapf**_**mic.mac.*j***_ is the total annual input of macro- and microplastics (mac, mic) to rivers from agricultural plastic films (apf) in sub-basin (*j*) (kg/yr);

**RSdif**_**mic.apf.*j***_**and RSdif**_**mac.apf.*j***_ are the annual input of microplastics (mic) and macroplastics (mac) to rivers from agricultural plastic films (apf) in sub-basin (*j*), respectively (kg/yr);

**APF***_**j**_* is the annual application amount of agricultural plastic films (mulching and greenhouse plastics) in sub-basin (*j*) (kg/yr);

**WSdif**_**mic.apf.*j***_ is the annual input of microplastics (mic) to cropland from agricultural plastic films (apf) in sub-basin (*j*) (kg/yr);

**WSdif**_**mac.apf.*j***_ is the annual input of macroplastics (mac) to cropland from agricultural plastic films (apf) in sub-basin (*j*) (kg/yr);

**MF***_**j**_* is the mechanical abrasion factor of microplastics from the agricultural plastic films in sub-basin (*j*) (unitless). We took the averaged mechanical value for polyethylene (PE) and poly(vinyl chloride) (PVC) based on the experimental study of Ren et al.^33^ Details are presented in Table S4.

**fr**_**residue.*j***_ is the residue rate of macroplastics in soils (0–1). This residue rate is based on the study of Zhang et al.^35^ which considers the collection and recycling of greenhouse plastics and mulching (Figure S2, Tables S3 and S4).

**fr**_**deg.*j***_ is the fraction of macro- and microplastics that are degraded (deg) in soils in sub-basin (*j*) (0–1). Details are in Tables S3 and S4 and Figure S1.

**FEsr***_**j**_* is the export fraction of macro- and microplastics from land to rivers via runoff (sr) in sub-basin (*j*) (0–1). This fraction is calculated as the 30 years’ averaged runoff divided by the 30 years’ averaged precipitation per sub-basin, following the approach of Zheng et al.^36^ (see details in Tables S3 and S4).

### 2.4 Inputs of Macro- and Microplastics to Rivers from Urbanization-Related Sources

We distinguished two types of sources for macro- and microplastics in rivers from urbanization-related sources (Figure 1, Table S2). We did our calculation in two steps. *First*, we calculated microplastics entering rivers from sewage systems. Sewage systems collect wastewater from the urban and rural populations. This wastewater goes to treatment facilities. After treatment, sewage effluents enter rivers. These effluents contain microplastics (7). This was done by integrating information from the modeling approaches of van Wijnen et al.^18^ Strokal et al.^17,19^ and Siegfried et al.^32^ Details of data sources and data processing can be founded in Tables S3 and S4.

*Second*, we calculated macro- and microplastics entering rivers from mismanaged solid wastes based on the data of Lebreton and Andrady.^6^ Details of our data processing are presented in Table S4. Then, we followed the approaches of Strokal et al.,^19^ van Wijnen et al.,^18^ and Siegfried et al.^32^ to calculate the transport of macro- and microplastics to rivers. This was done as a function of microplastic releases from macroplastics, residence time, sub-basin characteristics (e.g., slow and fast fraction), and the fraction of macroplastics entering rivers (8–18). The residence time, fast and slow fractions reflect the transportation ability and fragmentation of macro- and microplastics in the environment. In general, we implicitly account for two pathways of microplastic export from land to rivers following the approach of van Wijnen et al.^18^ The release rate of microplastics from the fragmentation of macroplastics is based on two fractions: slow and fast. The slow fraction has a longer residence time of microplastic release because macroplastics can be retained among river banks and it takes time for microplastics to release from macroplastics. The fast fraction has a shorter residence time of microplastic release because these macroplastics can easily be exported from sources to river systems, and these macroplastics are not stuck along river banks for a long time. The residence time depends on the drainage land area of the rivers. We followed the approach of Strokal et al.^19^ to calculate the residence time at the sub-basin scale.

Below, we list the equations to quantify the annual input of macro- and microplastics to rivers from urbanization-related sources. The data sources of our model inputs and data processing are presented in Supplementary Information Tables S3 and S4.
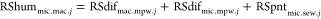
6
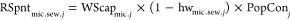
7
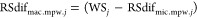
8
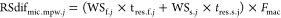
9
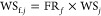
10
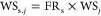
11
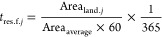
12
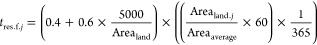
13
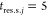
14
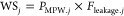
15
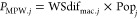
16
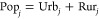
17
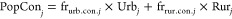
18where,

**RShum**_**mic.mac.*j***_ is the annual input of macro- and microplastics (mac, mic) to rivers from human waste in sub-basin (*j*). It consists of diffuse (mismanaged solid waste) and point sources (sewage systems) of plastics in rivers (see 7–9);

**RSpnt**_**mic.sew.*j***_ is the annual input of microplastics (mic) to rivers from sewage systems (sew) in sub-basin (*j*) (kg/yr). It is a point source of microplastics in rivers;

**RSdif**_**mac.mpw.*j***_ and **RSdif**_**mic.mpw.*j***_ are the annual input of macroplastics (mac) and microplastics (mic) to rivers from mismanaged solid waste in sub-basin (*j*), respectively (kg/yr);

**WScap**_**mic.*j***_ is the consumption rate of microplastics (mic) per capita (cap) in sub-basin (*j*) (kg/cap/yr). This model input is estimated based on the Provincial Chinese Human Development Index (HDI) (see details in Tables S3 and S4);

**hw**_**mic.sew.*j***_ is the annual removal fraction of microplastics (mic) during sewage treatment (sew) in sub-basin (*j*) (0–1). Treatment levels include primary, secondary, and tertiary treatments (see details in Tables S3 and S4);

**PopCon***_**j**_* is the total population that is connected to sewage systems in sub-basin (*j*), which includes the urban and rural population(people/yr) (18);

**WS***_**j**_* is the annual input of mismanaged solid waste in sub-basin from the total population (*j*) (kg/yr);

**WS**_**f.*j***_ is the annual input of macroplastics into a fast fraction in sub-basin (*j*) (kg/yr);

**WS**_**s.*j***_ is the annual input of macroplastics into a slow fraction in sub-basin (*j*) (kg/yr);

***t***_**res.f.*j***_ is the average residence time of macroplastic in the fast fraction in sub-basin (*j*) (yr). If sub-basins direct drain into the coastal waters and/or the land area is larger than 5000 km^2^, then **t**_**res.f**_ is estimated following 12 instead of 13;

***t***_**res.s.*j***_ is the average residence time of macroplastic in the slow fraction (yr) in sub-basin (*j*);

***F***_**mac**_ is the release rate of microplastics from macroplastics (0–1);

**FR**_**f**_ is the share of mismanaged solid waste with a fast fraction (0–1);

**FR**_**s**_ is the share of mismanaged macroplastic waste with a slow fraction (0–1);

**Area**_**land.*j***_ is the total land area of sub-basin (*j*) (km^2^);

**Area**_**average**_ is the average land area of the 50 largest river basins globally (km^2^);

***P***_**MPW.*j***_ is the amount of mismanaged plastic waste in sub-basin (*j*) (kg/yr);

***F***_**leakage.*j***_ is the fraction of macroplastic that can reach rivers in sub-basins (*j*) (0–1). This parameter is assigned to sub-basins based on solar radiation, soil pH, and also soil organic matter;

**WSdif**_**mac.*j***_ is the production of macroplastics (mac) per capita in sub-basin (*j*) (kg/cap/yr);

**Pop***_**j**_* is the total population in sub-basin (*j*) (people/yr);

**Urb***_**j**_* is the urban population in sub-basin (*j*) (people/yr);

**Rur***_**j**_* is the rural population in sub-basin (*j*) (people/yr);

**fr**_**urb.con.*j***_ is the fraction of the urban population connected to sewage systems (0–1);

**fr**_**rur.con.*j***_ is the fraction of the rural population connected to sewage systems (0–1).

### 2.5 Pollution Hotspots

We defined “pollution hotspots” for the annual input of macro- and microplastics in rivers inspired by studies of Wang et al.^37^ and Li et al.^38^ We ranked sub-basins according to the total amounts of macro- and microplastics in rivers per km^2^ of the sub-basin area per year from the lowest to the highest values. We used statistical quantiles (25, 50, 75, 100%) to define intervals for annual input of macro- and microplastics in rivers per km^2^ of the sub-basin from Level I (low inputs to rivers) to Level IV (high inputs to rivers) and Level V (very high inputs to rivers). This implies the following ranges for macro- and microplastics in rivers (kg/km^2^/yr): 0–0.044 (Level I), 0.044–1.5 (Level II), 1.5–100 (Level III), 100–160 (level IV), over 160 (level V). Levels IV and V are considered pollution hotspots in this study because their inputs of plastics to rivers per km^2^ are much higher than in other sub-basins (Level I–III non-hotspots).

## 3 Results

### 3.1 Pollution Hotspots of Macro- and Microplastics in Rivers

**Figure 2:**
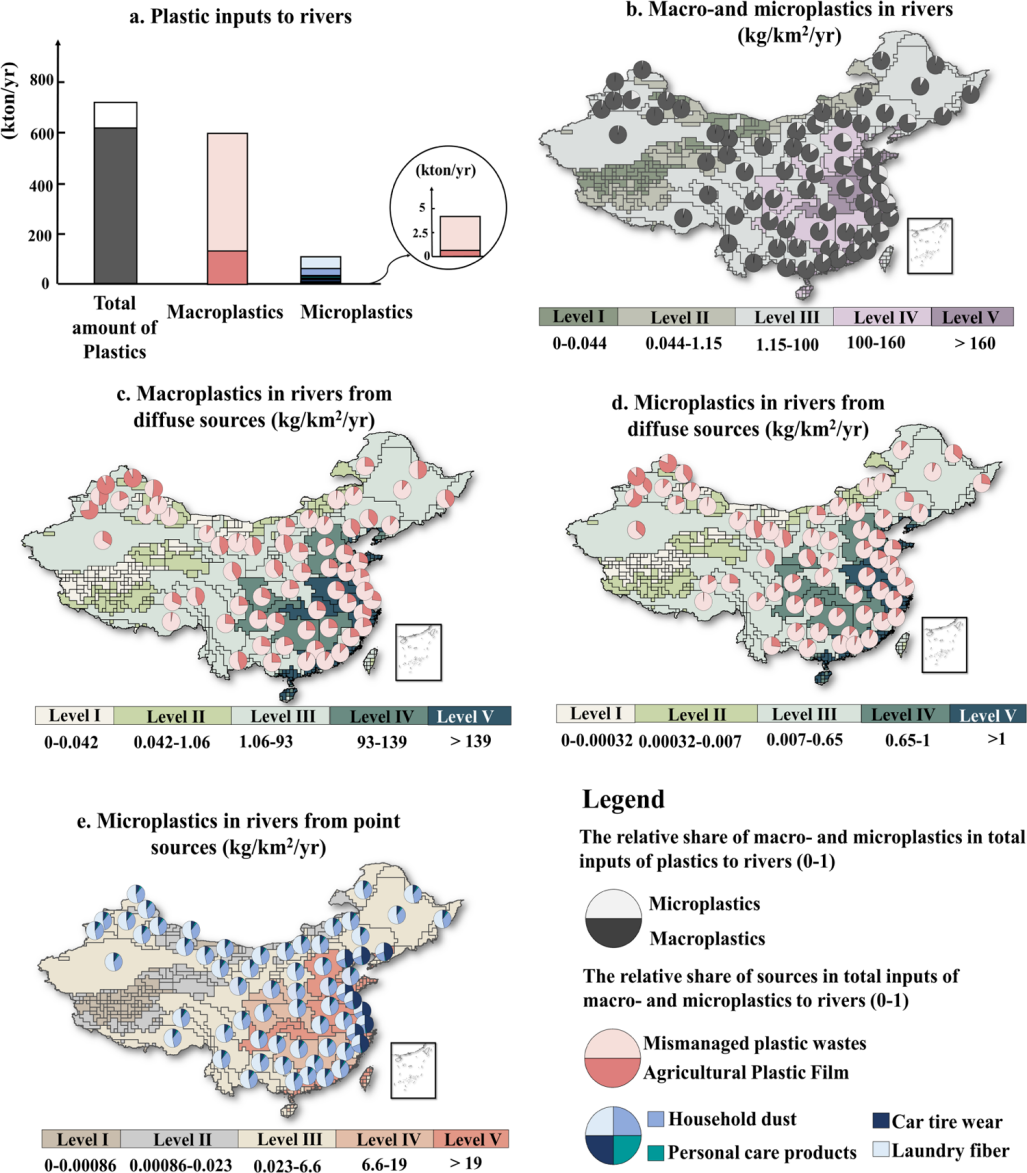
Inputs of macro- and microplastics to rivers in China in the year 2015 and their sources in terms of the contribution of crop production, and human activities in rural and urban areas. (a) Total national pollution level (kton/yr). (b–e) Inputs of macro- and microplastics to rivers and their sources at the sub-basin scale (kg/km^2^/yr). Five levels are distinguished from low (Level I) to high (Level V) for the annual input of plastics into rivers in kg/km^2^/yr. Sub-basins of Levels IV and V are defined as pollution hotspots because their rivers receive much higher inputs of plastics per km^2^ than rivers in the sub-basin of Levels I–III. Point sources refer to microplastics from sewage systems. Diffuse sources include macro- and microplastics from mismanaged solid wastes and agricultural plastic films. Source: The MARINA-Plastics model (China-1.0); see the model descriptions in the Materials and Methods section.

**Figure 3:**
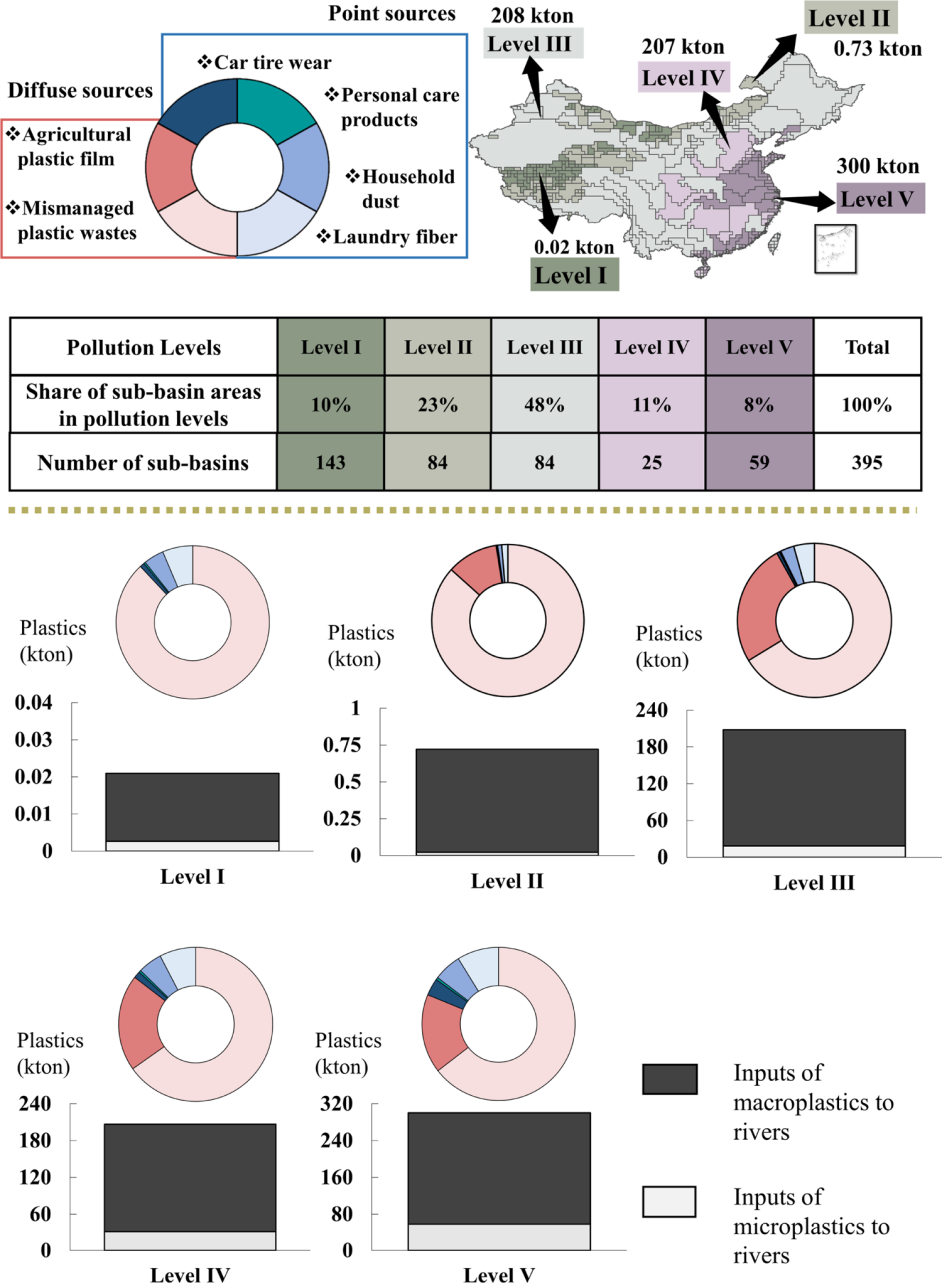
Sources of macro- and microplastics in rivers at five pollution levels for the year 2015 (kton/yr). Five pollution levels are defined based on inputs of plastics to rivers (note levels IV and V) are considered pollution hotspots (Materials and Methods section). The bar chart shows the inputs of macroplastics (black) and microplastics (gray) to rivers in sub-basins. Pie charts show the share of different sources including crop production and urbanization in river pollution with plastics. Crop production contributes macro- and microplastics in rivers from agricultural plastic films that are resulted from mulching and greenhouses (diffuse sources). Urbanization-related sources contribute macro- and microplastics in rivers from sewage systems (containing microplastics from laundry fibers, car tire wear, household dust, and personal care products; point sources), and mismanaged solid waste (containing macroplastics; diffuse sources). Source: The MARINA-Plastics model (China-1.0) (see the model described in the Materials and Methodssection).

Plastic inputs to rivers in China were estimated to be 716 kton in 2015 (Figure 2a). Around 85% of this amount was macroplastics. The remainder was microplastics (Figure 2a). There was a large spatial variability in river pollution among the 395 sub-basins (Figure 2b). Sub-basins in Levels I–II received less than 1.15 kg of plastics/km^2^/yr (Figure 2b), resulting in 0.02 kton for Level I and 0.73 kton for Level II (Figure 3). Together, these sub-basins cover 33% of the total study area and are located in the western and northern parts of China (Figure 3). For sub-basins in Level III, rivers received between 1.15–100 kg of plastics/km^2^/yr (Figure 2b), resulting in 208 kton in total (Figure 3). This total amount was similar to the amount in the sub-basins of Level IV (Figure 3). Two main reasons can explain this. One of the reasons is that the drainage areas of Level III sub-basins are larger (48% of the study area, Figure 3) compared to the drainage area of Level IV sub-basins (11% of the study area, Figure 3). This implies that Level III sub-basins have more area with activities contributing to more loadings of plastics to rivers. The other reason is related to the intensity of human activities per km^2^ of land. Level III sub-basins are generally with fewer human activities per km^2^ compared to Level IV and V sub-basins. This leads to the fact that the plastic yield in Level III sub-basins (kg of plastics in rivers per km^2^ of sub-basin area) is lower than the plastic yield in Level IV sub-basins. Generally, in the sub-basins of Levels I–III, over 90% of plastics were macroplastics (Figure 3). The western sub-basins in Level III received more macroplastics in rivers, compared to other sub-basins (Figure 2b). This is because of poor waste management in these sub-basins.

Sub-basins with macro- and microplastic inputs in rivers that exceed 100 kg of plastics/km^2^/yr (Figure 2b) were considered plastic pollution hotspots (Levels IV and V). This resulted in total plastic inputs of 207 kton for Level IV and 300 kton for Level V to the rivers (Figure 3). These sub-basins are concentrated in central and eastern China, covering around one-fifth of the Chinese basin area generating around 71% of total plastics in rivers (Figure 3). In these pollution hotspots, over 80% of these amounts of plastics in rivers (as mass) were contributed by macroplastics (Figure 3). Microplastic pollution is also important for some individual eastern sub-basins in Level IV and V because of the large amount of sewage effluents discharging into rivers and intensive crop production (Figure 2).

### 3.2 Plastic Pollution from Crop Production

Agricultural plastic films constituted 20% of plastics in Chinese rivers (Figure 2a). This contribution is to the total plastics in all rivers of China. Our model results show that approximately 2600 kton of agricultural plastic films were used in China’s crop production in 2015 (Figure S3). After collection and retention in soils, around 6% of plastics entered the rivers. However, there is a large variability in macro- and microplastics in rivers among sub-basins (Figure 2b–e). The share of agricultural plastic films in river pollution also differs among macro- and microplastics. In general, agricultural plastic films contributed to a limited extent to microplastics in rivers (Figure 2a). In contrast, agricultural plastic films were more important for macroplastics, but this is dependent on the characteristics of sub-basins (Figures 3 and S4). In sub-basins that we considered as non-pollution hotspots (Levels I–III), the share of agricultural plastic films to river pollution ranged from zero to 26% depending on the characteristics of the sub-basins (Figure 3).

**Figure 4:**
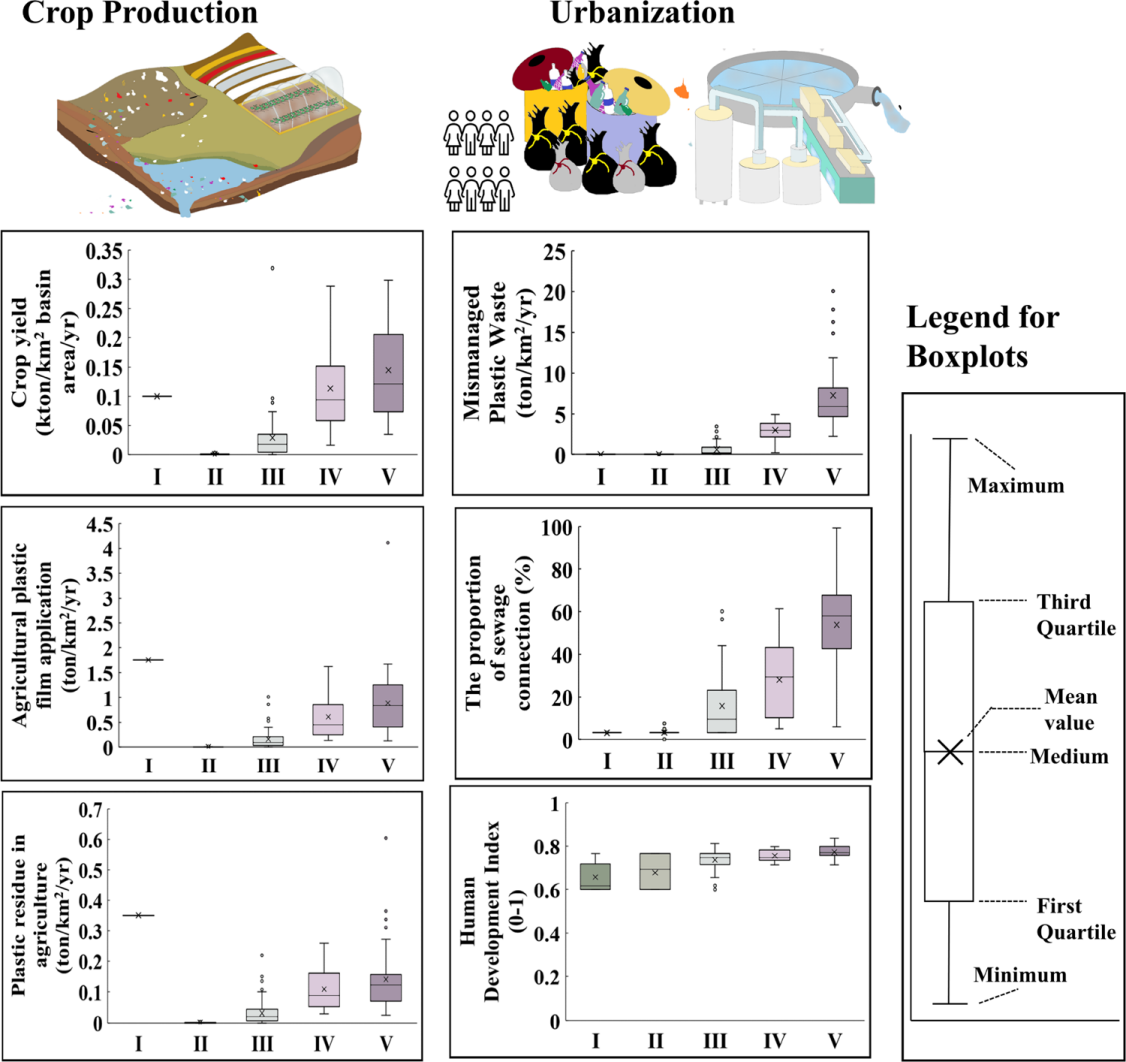
Boxplots of the main drivers of macro- and microplastic pollution in rivers from crop production and urbanization-related sources for the year 2015. The drivers are analyzed for the sub-basins and are classified based on the five pollution levels (see the Materials and Methodssection). Drivers for crop production include crop yield (kton/km^2^ basin area/yr), applications of agricultural plastic films including mulching and greenhouses (ton/km^2^/yr), and plastic residues in cropland (plastics that are left in soils after collection, ton/km^2^/yr). Drivers for urbanization include the production of mismanaged solid waste (ton/km^2^/yr), the proportion of the population connected to sewage systems (%), and the Human Development Index (HDI, 0–1). The human development index includes the years of schooling, Gross National Income per capita, and life expectancy per year. Source: The MARINA-Plastics (China-1.0 model) (see the model described in the Materials and Methodssection).

For Level I–II sub-basins, the contribution of agricultural plastic films to plastics in rivers was limited. For Level I sub-basins only a limited amount of agricultural plastic films were applied and stayed in the soil, therefore, agricultural plastic films did not contribute to plastics in their rivers (Figure 3). For Level II sub-basins, agricultural plastic films contributed 11% to plastics in their rivers, which was higher than the contribution for sub-basins in Level I. This is associated with the intensity of agricultural practices. Limited agricultural activities were presented in sub-basins of Levels I–II. For example, these sub-basins had a crop yield of 0.004 kton/km^2^/yr and the application of agricultural plastic films is around 0.06 ton/km^2^/yr (statistical mean values, Figure 4). After crop harvesting, agricultural plastic films were largely collected. Plastic residues in soils for these sub-basins were calculated at 0.012 ton/km^2^/yr (a statistical mean value, Figure 4).

For Level III sub-basins, the contribution of agricultural plastic films to plastics in rivers was more than doubled compared to sub-basins in Levels I–II (Figure 3). This is because the Level III sub-basins had a large land area and intensive crop production there. A crop yield was estimated to be at 0.03 kton/km^2^/yr (a statistical mean value). The use of agricultural plastic films (0.16 ton/km^2^/yr on average) was higher than in the sub-basins of Levels I–II (Figure 4). As a result, plastic residues in soil of the Level III sub-basins were higher compared to that in the sub-basins of Level I–II. A large share of agricultural plastic films to river pollution was estimated for individual northwest sub-basins of Level III, where agricultural plastic films were responsible for more than half of the macro- and microplastics in rivers (Figure 2c,d) because of intensive agriculture there.

For the sub-basin of Level IV–V (hotspots), the share of agricultural plastic films in river pollution ranged from 17% to 20%. This was associated with the higher use of plastic mulching and greenhouses for crop production in these sub-basins, where plastic film collection and recycling were limited (Figure 3). Although the share of agricultural plastic films in Levels IV–V sub-basins was lower than in the Level III sub-basins, the plastic production in Levels IV–V sub-basins are higher than in the Level III sub-basins. This is because Levels IV–V sub-basins are urbanized, thus, more plastic pollution from urbanization-related sources, rather agriculture. However, agriculture is more intensified in the Level IV–V sub-basins, compared to the other sub-basins. In the Levels IV–V sub-basins, crop yield and the use of agricultural plastic films were calculated at 0.13 kton/km^2^/yr and 0.79 ton/km^2^/yr, respectively (statistical means for the sub-basins, Figure 4). Plastic residues in soils are 0.13 ton/km^2^/yr (a statistical mean value, Figure 4). These residues were higher than in the non-hotspots (Levels I–III).

### 3.3 Plastic Pollution from Urbanization-Related Sources

Urbanization-related sources constituted 80% of total plastics in rivers (Figure 2a). Mismanaged plastic waste from the total population was the major source of macroplastics in rivers (Figure 3). Sewage systems were the major sources of microplastics in rivers (Figure 2a,e, Supplementary Figure S5). Our model results show that mismanaged solid waste contributed 65% to macro- and microplastics in all rivers of China in 2015 (Figure 2a). For macroplastics alone, the contribution of mismanaged solid wastes was 76% of the total amount of macroplastics (Figure 2a). Sewage effluents discharged around 103 kton of microplastics into Chinese rivers. Laundry fibers and household dust in sewage effluents take the dominant share in point source pollution for almost all sub-basins (Figure 2e). Among sub-basins (Level I–V), the contribution of plastics from sewage systems and mismanaged solid waste varied depending on urbanization.

For Level I–II sub-basins, mismanaged solid wastes contributed by over 85% to plastics in their rivers, and macroplastics were dominated. Sewage systems had a limited contribution to plastics in their rivers (2–12% Figure 3). This is associated with low urbanization and societal developments in these sub-basins. The sub-basins of Level I–II had a low population density (Figure S6). The human development index was around 0.67 (Figure 4). Only 3% of the population was connected to sewage systems and the production of mismanaged solid wastes was estimated at around 0.005 ton/km^2^/yr (a statistical mean over the sub-basins; Figure 4).

For Level III sub-basins, mismanaged solid wastes contributed by over 60% to plastics in their rivers. Sewage inputs of microplastics accounted for 8% of plastics in these sub-basins. However, rivers in Level III received much more plastics from urbanization-related sources, compared to Level I–II sub-basins. The differences can be explained by better societal developments in Level III sub-basins (Figure S6). The human development index was around 0.74 on average (Figure 4). Compared to sub-basins in Levels I–II, the sub-basins of Level III were more populated with more people connected to sewage systems (around 16% of the total population), and more production of mismanaged solid wastes (e.g., 0.6 ton/km^2^/yr on average, Figure 4).

For Levels IV–V sub-basins (hotspots), mismanaged solid waste contributed around 65% to plastics in rivers (Figure 3). This is because these sub-basins had insufficient waste collection and management (Figure 2b,c). They were the most populated and urbanized areas among sub-basins (Figure 4). The human development index in Levels IV–V sub-basins was around 0.77 (statistical average). The production of mismanaged solid waste in the sub-basins of Level IV and V was much higher than in the other sub-basins: around 2.9 ton/km^2^/yr for Level IV and 7.2 ton/km^2^/yr for Level V (Figure 4). More microplastic inputs to rivers are calculated for hotspot sub-basins (Level IV–V) compared to non-hotspot sub-basins (Level I–III). Sewage systems were responsible for 15–18% of plastics in rivers (Figure 3, Levels IV–V). This is the result of a large share of the population (over 44% of the total population) connected to sewage systems. For Level V sub-basins, car tire wear in sewage effluents was the dominant source of microplastics in rivers (around 50%). This is because of densely populated cities with high-level economic developments in these sub-basins (Figure 4).

## 4 Discussion

### 4.1 Model Evaluation and Uncertainties

Validating our model for macroplastics and microplastics in 395 sub-basins of China is challenging. Observational data are scarce, and often presented as plastic particles and limited in time and space. In addition, our model calculates the mass of plastics, making the comparison with experiments (e.g., particles) difficult. Therefore, we evaluated our model by adopting a widely used “building trust” approach for large-scale water quality models.^17,39^ In our study, this approach includes five options to build trust in our model:

*Option 1* is to evaluate model outputs against existing studies. Our model outputs are in line with available studies (Table S6). Wang et al.^21^ and our study indicate that the middle and eastern parts of China received considerable amounts of microplastics in rivers. We estimated 103 kton of microplastics in Chinese rivers from sewage systems. This is somewhat lower than in Wang et al.,^21^ because of differences in considered pollution sources. Our model calculates a large contribution of sewage systems to microplastic pollution in rivers of mainland China, which is in line with Cheung et al.^40^ Ren et al.^34^ estimated the amount of microplastics entering rivers from soil erosion based on soil sampling data in 19 Chinses provinces. They showed that pollution hotspots are located in central and eastern China. Our hotspots of microplastic pollution from agriculture are comparable with their findings (Figure S4). We show that the downstream part of the Yangtze basin is a hotspot of plastic pollution, which is in line with the observation of Han et al.^41^

*Option 2* is to compare model inputs with existing studies. We estimated that around 16700 kton of mismanaged solid waste was produced in China in 2015 (Figure S3). This is higher than in Li^42^ and Jambeck et al.^29^ in 2010 because of differences in modeling approaches, datasets, and time scales. Our estimation for plastic residues in the soil is partly in the range of local experimental results (Table S7). This is because temporal differences and experiments often cover specific fields, whereas our study is at the sub-basin scale. Moreover, the spatial distribution of our plastic residues in agricultural soils is comparable with the spatial distribution of the second soil census^35^ (Figure S7). The slight differences can be explained by differences in temporal scales and approaches differences between our study and the soil census. Data are lacking for some western, southern, and eastern China in the soil census report, but both of our results indicate that the central and northeastern sub-basins are hotspots of plastic pollution. We estimated a high runoff fraction in southern and eastern sub-basins in China, which is comparable with the runoff fraction at the basin scale (from the Year Book of China Water Resources)^43^ (details see Table S4).

*Option 3* is to build trust in our model based on experts’ knowledge of uncertain model parameters. Our model is an integration of the existing knowledge and the literature on soil–water interactions for plastics. We reviewed relevant literature on the physical, chemical, and biological processes influencing plastic degradation^44−46^ (Table S5, Figure S1). Moreover, we verified our estimation of the mechanical abrasion fraction by considering the effects of solar radiation based on experts’ knowledge. High solar radiation is associated with a higher mechanical abrasion fraction and vice versa. Details of assigned mechanical abrasion fractions in sub-basins were presented in Table S4. We used existing knowledge in the field of soil erosion to quantify macro- and microplastic transport from agricultural land to rivers by surface runoff.^36,47−49^

*Option 4* is to perform a sensitivity analysis. We did a sensitivity analysis for microplastics, macroplastics, and total plastic inputs to rivers, respectively (Figure S8). We tested the sensitivity of model outputs to changes in model inputs. We selected 13 model inputs which were increased and decreased by 10%, following the approach of Strokal et al.^19^ As a result, we had 26 alternative model runs. We compared the model outputs between the original model run (presented in Section 3) and alternative model runs (from sensitivity analysis). Our model outputs are relatively sensitive to changes in some of the model parameters (Supplementary Information Figure S8). For example, model outputs are calculated to increase or decrease by 0.3% as a result of a 10% increase or decrease in the degradation rate of macro- and microplastics from agriculture-related sources. Around 3% increases or decreases are calculated for model outputs as a result of a 10% increase or decrease in the removal fraction of microplastics in sewage systems from urbanization-related sources. Generally, we calculated higher changes in model outputs due to changes in the fraction of the population connected to sewage systems and in the urban and rural population. Our sensitivity analysis shows the important model inputs influencing model outputs. This can contribute insights into plastic management on land.

*Option 5* is to reflect on the uncertainties and limitations in the model structure. As an integrated model, our model has uncertainties and limitations related to the model structure and assumptions. Our model takes a lumped approach. The model is developed for large-scale analyses. We scaled up the main processes into several model parameters and inputs for sub-basins. Our study calculated the mass of plastics in rivers, we did not account for the changes in the size and weight of plastic particles during the transport process. This differs from the study of Ren et al.^34^ They calculated the microplastic pollution in agriculture based on the assumptions for the size and weight of particles in the soil. In addition, we did not consider the variability within the sub-basins (e.g., the distance from cropland to the river).^5^ Thus, our study should not be applied to analyzing local situations (e.g., specific fields or cropland). This differs from the study of Meijer et al.^5^ because they used a probabilistic modeling approach to estimate the probability of microplastics transport from one cell to the other (3 × 3–arc sec scale). We calculated macro- and microplastics entering rivers via runoff as inspired by the approach of Zheng et al.^36^ which is used for nutrients. Runoff implicitly reflects the impact of land use and slope on plastic export. The study of Meijer et al.^5^ used a different approach to account for microplastic transport from land to rivers, which quantified the effects of slope to plastic transportation. We believe the uncertainties in our modeling approach will not largely affect our main findings, because we considered the important processes of plastic transport associated with application rates, in-soil degradation and fragmentation, and surface runoff.

In our model, we considered sewage inputs of microplastics to rivers from car tire wear, personal care products, laundry fiber, and household dust. These are point sources of river pollution. However, microplastics from car tire wear can also enter rivers with road runoff,^50^ which are diffuse sources of river pollution. These diffuse sources are not considered in our model. Another limitation is that the model does not consider wind drift which can potentially influence plastic transport from soil to rivers and increase plastic pollution.^49,51,52^ We also realized there are more missing sources, for instance, soil erosion,^34,49^ industries and ships,^21,53^ sludge reuse and organic fertilizer utilization,^44,54^ floods,^49,51,55^ and stormwater.^56^ Thus, our river pollution levels might be underestimated. Nevertheless, we believe that our conclusions do not change because we focus on crop production and urbanization impacts on macro- and microplastic pollution. Our focus covered the important activities (e.g., mismanaged solid waste, sewage, and agricultural plastic film) in China.^21,29,35^ Our study is presented at an annual step. However, the seasonality of plastic pollution has been found in existing studies, which are related to runoff and discharge load.^57,58^ In the next steps, future research can build on this and include other aspects (e.g., missing sources and seasonality).

We are aware of the limitations and uncertainties in our assumptions for model inputs. Mismanaged solid waste is one of the important model inputs. We used the data for this input from Lebreton and Andrady.^6^ To our knowledge, it is the most complete dataset that covers China as a whole and is at the grid of 0.5°. This resolution enables us to aggregate the data into sub-basins for China. However, we realized that the data might have some uncertainties associated with, for example, collection rates of waste, and its management. The uncertainty is also related to our assumptions on microplastics entering soil via the mechanical process. We are aware that part of agricultural plastic films may be buried in the soil during farming practices.^59^ Thus, we may overestimate the microplastics in the soil caused by mechanical processes. However, these limitations may not affect our conclusion on the spatial differences of environmental and hydrological factors (e.g., solar radiation, pH, runoff) as well as sub-basin characteristics (e.g., population, sewage connections, mismanaged waste) because of our focus on the sub-basin (large geographical units) and national analyses.

Our results are for the year 2015 because it is a representative year. More and more policies and action plans have been published to tackle plastic pollution from both agriculture and daily practices after 2014 (Table S1). In addition, there are fewer changes between 2015 to 2020 in plastic management based on the data from China Statistic Yearbook.^60,61^ For instance, the application amount of agricultural plastic films decreased by 8% in 2019 compared to 2015.^60,61^ The population only increased by 2% between 2010 to 2020^62,63^ (see Supplementary Figure S6). Thus, we believe our modeled results are still useful for public to understand the current plastic pollution. We expect that pollution levels are even higher today than in 2015. This means that our conclusions on pollution hotspots and their causes are still relevant for plastic management today.

### 4.2 Insights into Plastic Pollution Reduction

**Figure 5:**
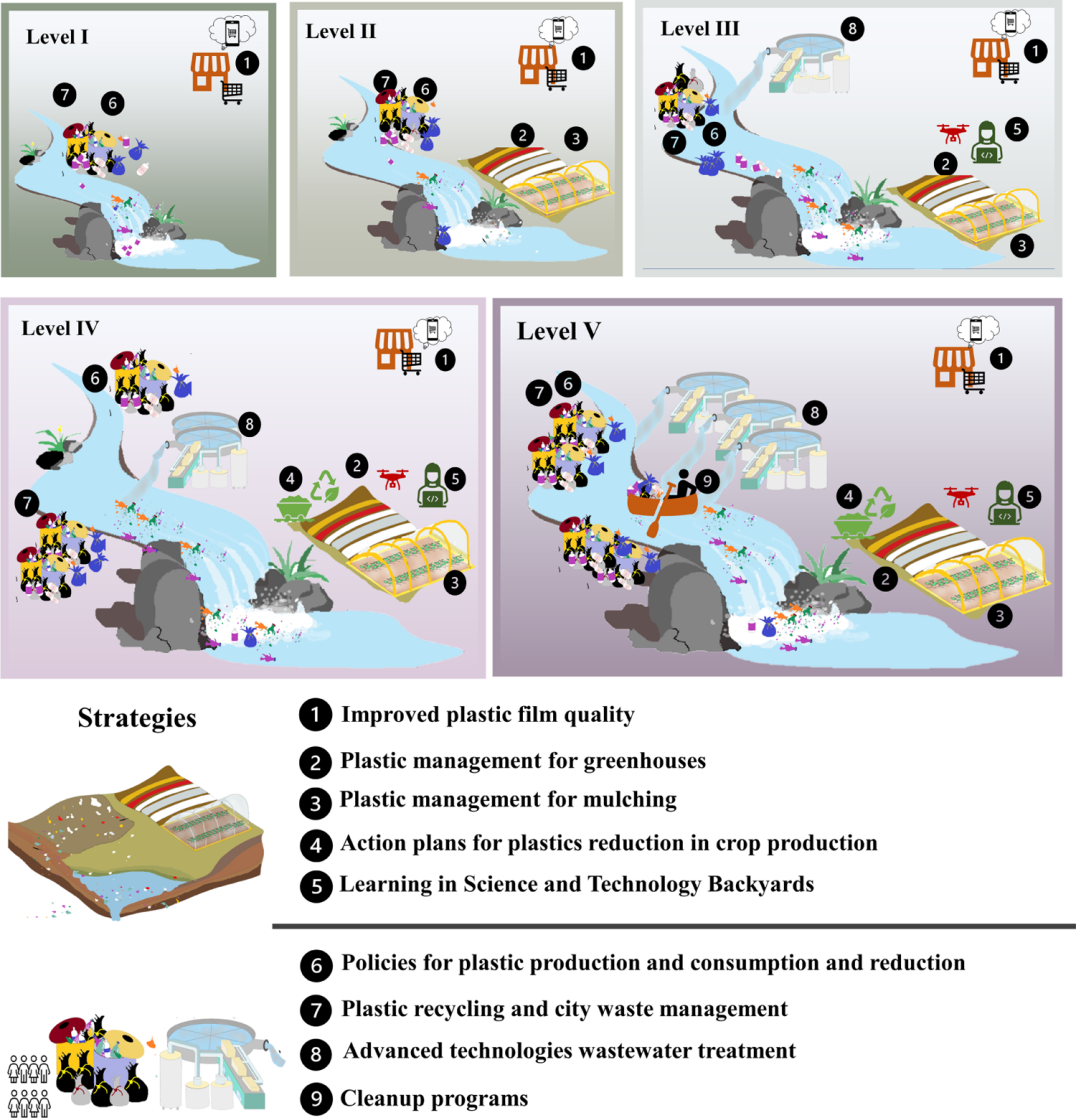
Nine strategies for plastic pollution reduction in Chinese sub-basins. Numbers 1 to 5 mitigate plastic pollution from agriculture. Numbers 6 to 9 mitigate plastic pollution from urban areas.

Our study improves our understanding of national macro- and microplastic pollution and its sources. Our findings could support the design of plastic reduction strategies in response to green development in China: sustainable agriculture, sustainable urbanization,^64^ and clean water. We calculated that macroplastics take the dominant share in the total plastic inputs to rivers. Most macroplastics in rivers are from mismanaged solid waste. This implies waste management is important for plastic pollution reduction in the future (Figure 5). For instance, strategies to better manage solid waste and agricultural plastic films could be implemented at the national level (Strategies 1, 2, 3, 6, 7 in Figure 5). It can include policies for effectively controlling plastic production, consumption, recycling, and treatment in urban and rural areas. Regulation on the management of agricultural plastic films aims to build a green agricultural environment by inspecting the plastic films to ensure their high quality for agricultural activities (e.g., the thickness of agricultural films should be over 0.02mm). Plastic recycling and waste management (e.g., better collection) could be improved in all sub-basins to reduce plastic pollution from mismanaged solid waste. Examples of such a strategy (Strategy 7 in Figure 5) could be an “Eco-industrial Park” to improve the waste systems^65^ and waste management in Singapore and Shanghai.^66,67^

Our study provides insights for a better understanding of plastics in rivers and their sources at the sub-basin scale. It can help to prioritize plastic reduction strategies in China. It indicates where (sub-basins) and what (pollution sources) efforts are needed to reduce plastic pollution (Figure 5). For example, we identified hotspot sub-basins (Level IV and V) that can be priority areas for plastic control. For instance, in sub-basins of Level IV and V, Strategies 1, 2, 3, 6, 7, extra Strategy 4 (e.g., better agricultural films recycling in cropland), Strategy 5 (e.g., integrating scientific knowledge and farming practices in agricultural practices^68^), and Strategy 8 (e.g., using membrane bioreactors^69^) and Strategy 9 (different barriers for plastics collection, cleanup programs such as (e.g., https://theoceancleanup.com/)^70^) could be applied in these sub-basins (see more examples in Figure 5). For Level I sub-basins, reducing plastics from sewage systems and mismanaged waste (Strategies 1, 6, 7) might be beneficial because of the large contribution of these sources to water pollution. For Level II sub-basins, strategies 1, 2, 3, 6, and 7 may be considered (Figure 5). For Level III sub-basins, Strategies 1, 2, 3, 5, 6, could be implemented there. Our modeling approach can help to explore the possibilities for future plastic pollution reduction.

The current study of Borrelle et al.^71^ pointed out that efforts on plastic pollution mitigation may not reduce the growth in plastic waste. In the “14th Five-Year Plan” in China,^72^ plastic reduction has been proposed as one of the future targets. MARINA-Plastics (China-1.0) can be a tool for supporting the call for the “14th Five-Year Plan” in China. Our model can incorporate the target of the “14th Five-Year Plan” and existing technologies to explore the possibility of assessing the effects of plastic reduction in the future. From this, we can provide information on where to prioritize plastic pollution reduction (hotspots) in the future, and how to reduce plastic pollution by sources at different sub-basins in China. This information is important for designing new plastic management strategies in China. China is one of the biggest plastic consumers globally, and plastic pollution mitigation in China is important across the world. Our model can be applied to other regions that experience similar plastic pollution problems associated with crop production and urbanization.

Our study is the first attempt to account for macro- and microplastics from crop production and human activities in urban and rural areas. MARINA-Plastics model (China-1.0) is developed to quantify the macro- and microplastic pollution in 395 Chinese sub-basins. In 2015, 716 kton of plastic entered rivers causing plastic pollution. Approximately 20% of the basin area is located in central and eastern sub-basins, contributing around 71% of plastics in rivers. These sub-basins are densely populated with intensive agricultural activities. Agricultural plastic films are responsible for 20% of plastics in Chinese rivers and the remainder from other sources. Mismanaged waste from urban and rural is responsible for 65% of macro- and microplastics in rivers. The majority of microplastics in Chinese rivers are discharged from sewage effluents.

## Supporting Information Available

Estimated plastic degradation rate in soil (Figure S1), illustration of the area-weighted approach (Figure S2), additional results (Figures S3–S6), comparison of our model results with soil survey data (Figure S7), sensitivity analysis results (Figure S8), summary of existing policies related to plastic management in China (Table S1), source of plastic pollution (Table S2), sources of model inputs, data processing methods (Tables S3 and S4), summary of plastic degradation rate (Table S5), and comparison of our model results with existing studies (Tables S6 and S7) (PDF)
