# Supplementary material for: The Plastic Age: River Pollution in China from Crop Production and Urbanization
Source: Environ Sci Technol. 2023 Aug 1;57(32):12019–32. doi: 10.1021/acs.est.3c03374 (PMC10433511; doi:10.1021/acs.est.3c03374)
Supplement: Supplementary file 1 — es3c03374_si_001.pdf [file es3c03374_si_001.pdf]

Supplementary Information:

The Plastic Age: River Pollution in China from Crop Production and Urbanization

Yanan Li [a,b], Qi Zhang [a,b], Jantiene Baartman [c], Jikke van Wijnen [d], Nicolas Beriot [c], Carolien Kroeze [b,f], Mengru Wang [b], Wen Xu [a*], Lin Ma [e], Kai Wang [a], Fusuo Zhang [a], Maryna Strokal [b*]*

[a] College of Resources and Environmental Sciences; National Academy of Agriculture Green Development; Key Laboratory of Plant-Soil Interactions of MOE, China Agricultural University, Beijing 100193, China.

[b] Water Systems and Global Change Group, Wageningen University & Research, Droevendaalsesteeg 4, Wageningen, 6708 PB, The Netherlands

[c] Soil Physics and Land Management Group, Wageningen University & Research, Droevendaalsesteeg 3, Wageningen, 6708 PB, The Netherlands

[d] Department of Science, Faculty of Management, Science & Technology, Open University, Heerlen, 1081 HV, The Netherlands

[e] Key Laboratory of Agricultural Water Resources, Center for Agricultural Resources Research, Institute of Genetics and Developmental Biology, Chinese Academy of Sciences, 286 Huaizhong Road, Shijiazhuang 050021, China

[f] Environmental Systems Analysis Group, Wageningen University & Research, Droevendaalsesteeg 4, Wageningen, 6708 PB, The Netherlands

* Corresponding author: yanan.li@wur.nl; maryna.strokal@wur.nl; wenxu@cau.edu.cn;

Supplementary figures:

Figure S1. Estimated plastic degradation rates in the soil based on soil organic matter, pH, and solar radiation (greenish maps, 0-1) and the associated averaged degradation rates at the sub-basin scale in China (a yellowish map, 0-1).

Figure S2. A schematic overview of the area-weighted approach.

Figure S3. Plastics on land from mismanaged solid waste, and agricultural plastic films from crop production including mulching, and greenhouses at the sub-basin scale (maps, A: kg/km^2/yr; B: 10^5 kg/yr), and in China a whole (a table, Tg/yr) in the year 2015.

Figure S4. Inputs of macro-and microplastics to rivers from mismanaged plastic waste and agricultural plastic films from crop production including mulching and greenhouses at the sub-basin scale in China in the year 2015 (kg/km^2/yr).

Figure S5. Inputs of microplastics to rivers from car tire wear, household dust, personal care products, and laundry fiber in the year 2015 (kg/km^2/yr).

Figure S6. The spatial distribution of population in Chinese sub-basins in the year 2020 (10^4 people/yr and people/km^2/yr).

Figure S7.Comparison of our plastic residues with the soil survey data (10^5 kg/yr).

Figure S8. Results of the sensitivity analysis for the MARINA-Plastics (China-1.0) model.

Supplementary Tables:

Table S1. An overview of plastics-related policies or action plans in China.

Table S2. Sources of mcro-and microplastics in rivers from crop production and urbanization related sources.

Table S3. Sources of the data for model inputs.

Table S4. Descriptions of how agricultural model inputs are processed to the sub-basins for the MARINA-Plastics model (China-1.0).

Table S5. The fraction of plastic degradations in the soil as influenced by biological, physical, and chemical processes (0-1).

Table S6. Comparisons of our modeled microplastic inputs to rivers in Chinese sub-basins with other studies (kton/yr).

Table S7. Comparisons of our modeled microplastics in the soil from crop production with experimental data between 1999 and 2021 (kg/km^2/yr).

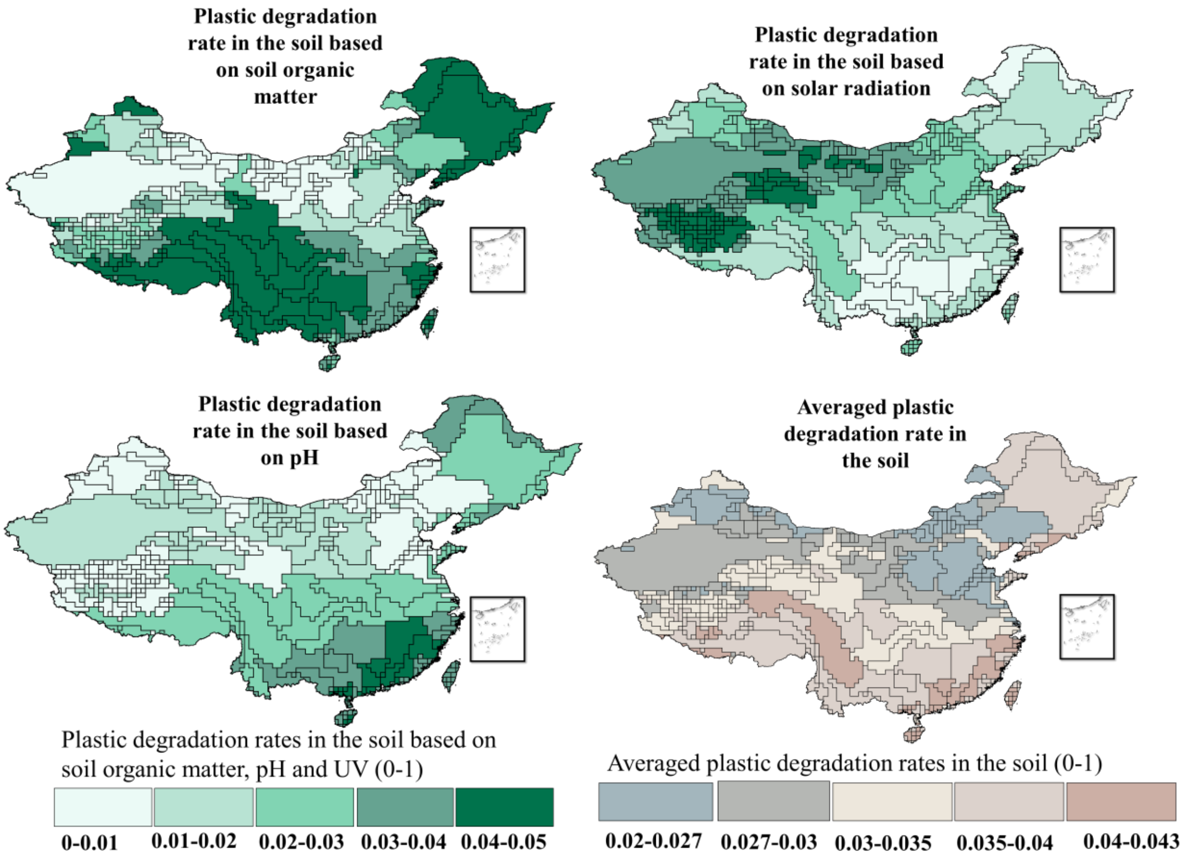

Figure S1. Estimated plastic degradation rates in the soil based on soil organic matter, pH, and solar radiation (greenish maps, 0-1) and the associated averaged degradation rates at the sub-basin scale in China (a yellowish map, 0-1). The average rates are estimated statistically using the degradation rates in the soils based on soil organic matter, pH, and solar radiation. The original information on soil organic matter and pH is from the National Earth System Science Data Center (NESDC) [1, 2]. Solar radiation data is from the GloWPa model (Global Waterborne Pathogen) [3]. The original data on plastic degradation rates are summarized in Table S3. The original soil organic matter data were available at the resolution of 1 km × 1km, solar radiation data were available at the resolution of 0.5-degree cells, and soil pH data were available at 90 m × 90 m. We aggregated this data into sub-basins as explained in Table S4.

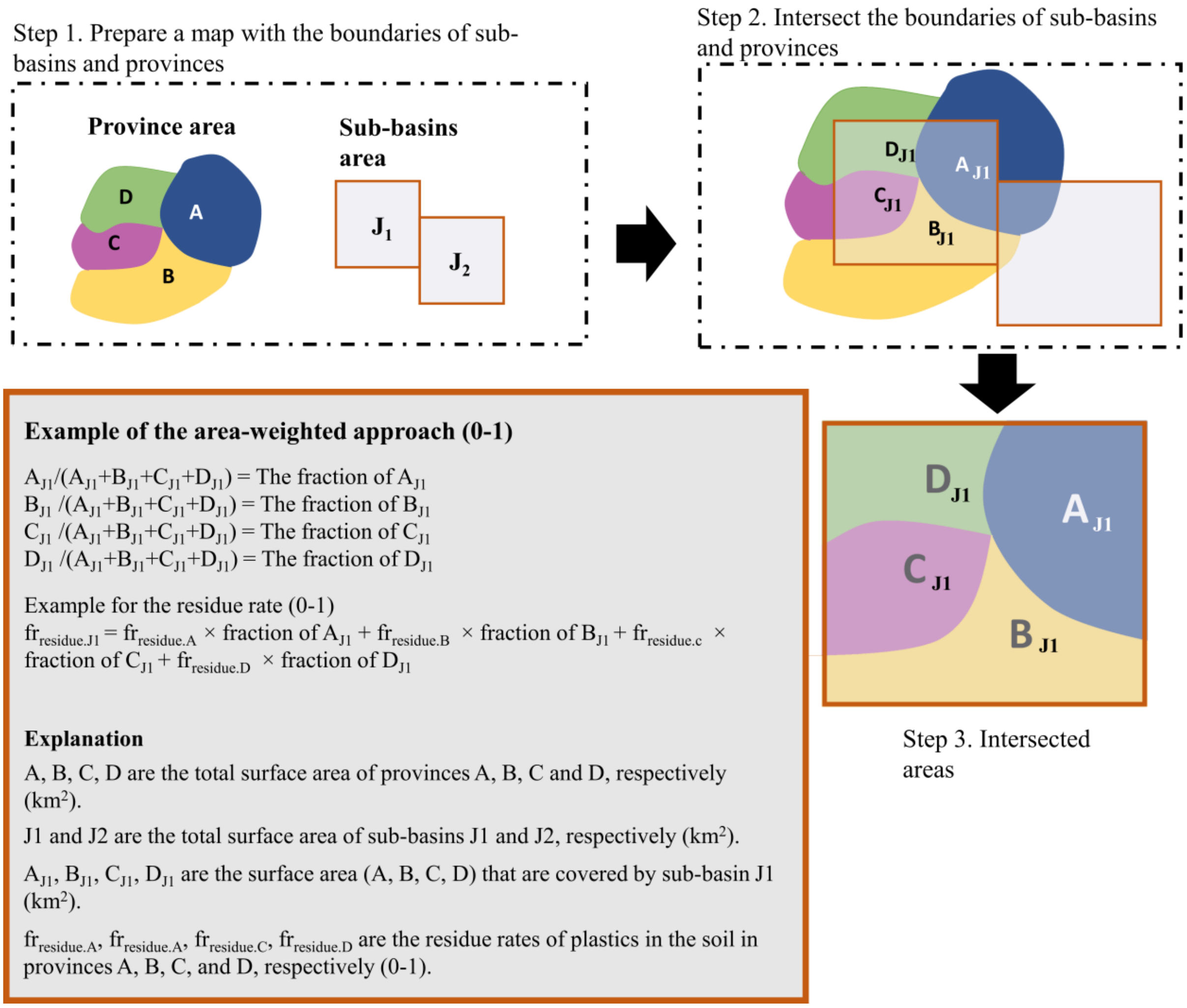

Figure S2. A schematic overview of the area-weighted approach. This approach is used to convert provincial data to a sub-basin scale. The grey box shows an example of how to convert residue rates data to a sub-basin scale. Details are in Table S4.

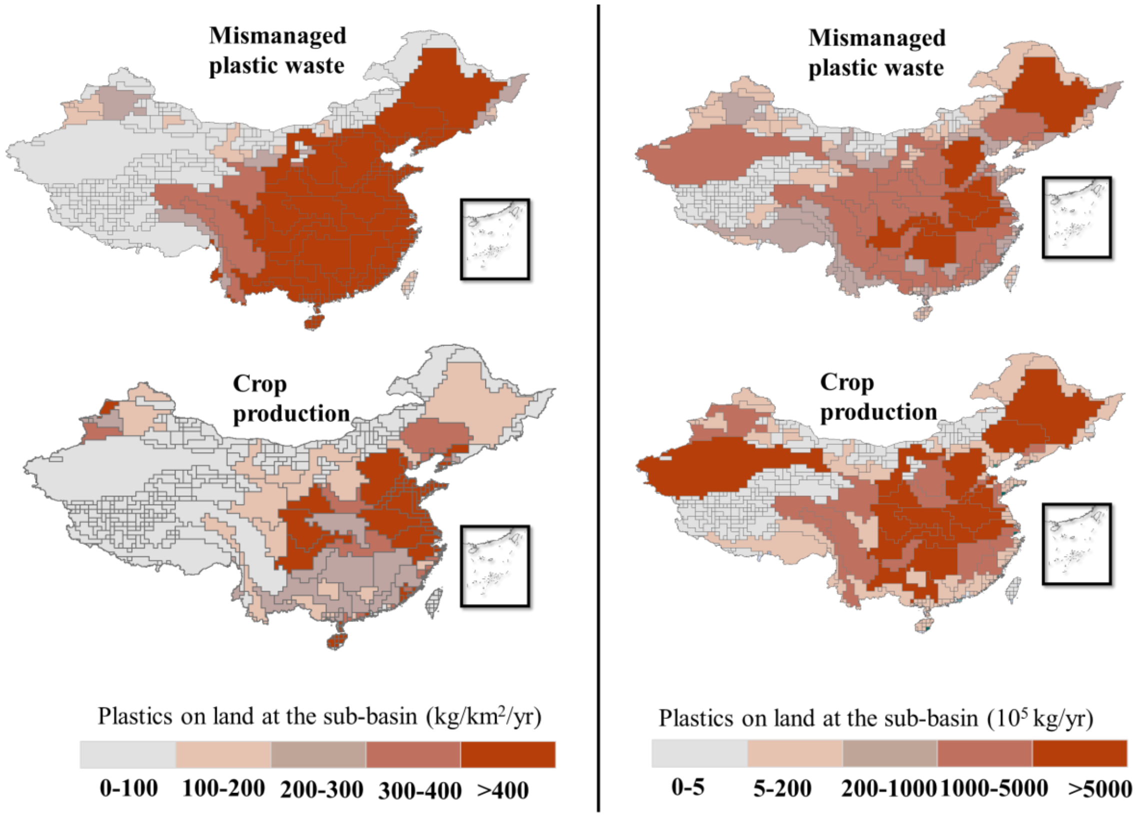

Total plastics on land in China from 395 sub-basins

**Mismanaged plastic waste**	**17 Tg/yr**
**Plastics from crop production (greenhouse and mulching)**	**2.6 Tg/yr**

Figure S3. Plastics on land from mismanaged solid waste, and agricultural plastic films from crop production including mulching, and greenhouses at the sub-basin scale (maps, A: $kg/km^2/yr$; B: 10^5 kg/yr), and in China a whole (a table, Tg/yr) in the year 2015. Source: the MARINA-Plastics model (China-1.0) for mismanaged solid waste and the China Statistic Yearbook for mulching and greenhouses. Raw data was aggregated into sub-basins.

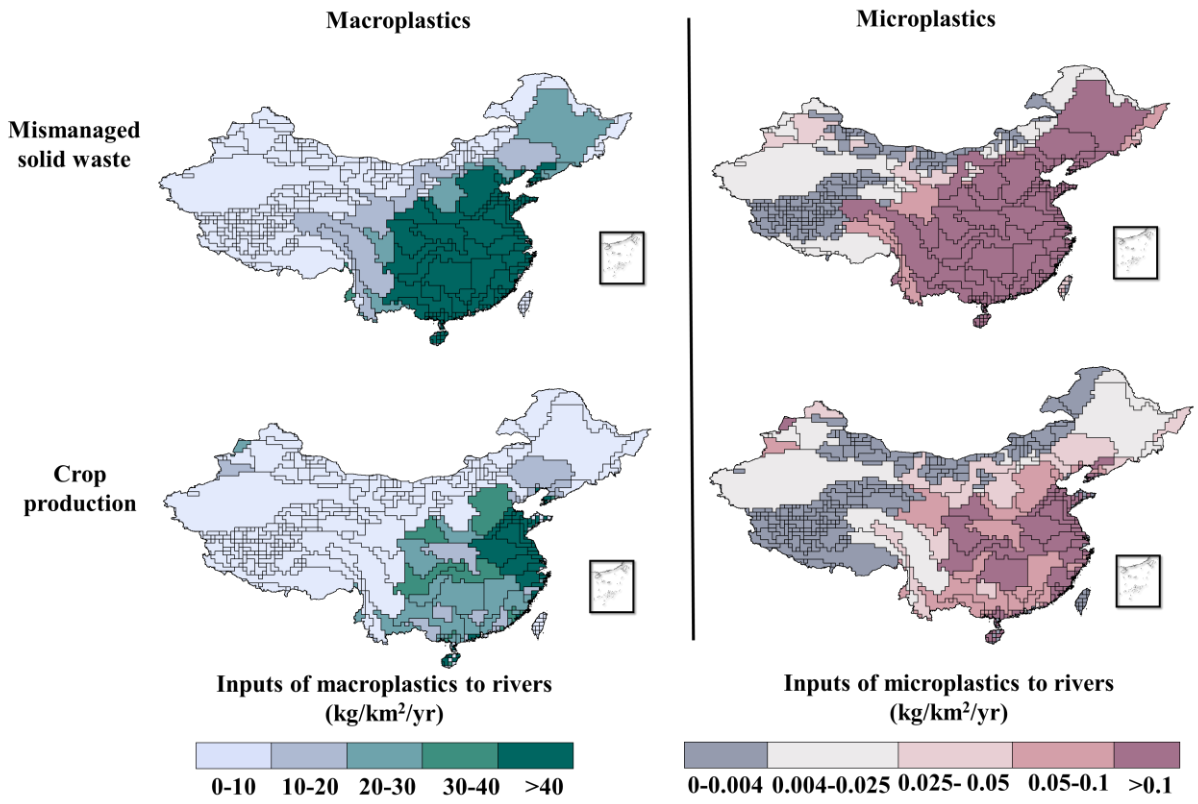

Figure S4. Inputs of macro-and microplastics to rivers from mismanaged solid waste and agricultural plastic films from crop production including mulching and greenhouses at the sub-basin scale in China in the year 2015 (kg/km^2/yr). Mulching and greenhouses are defined as agricultural plastic films in this study. Source: the MARINA-Plastics model (China-1.0), see the model described in the "Materials and Methods" section.

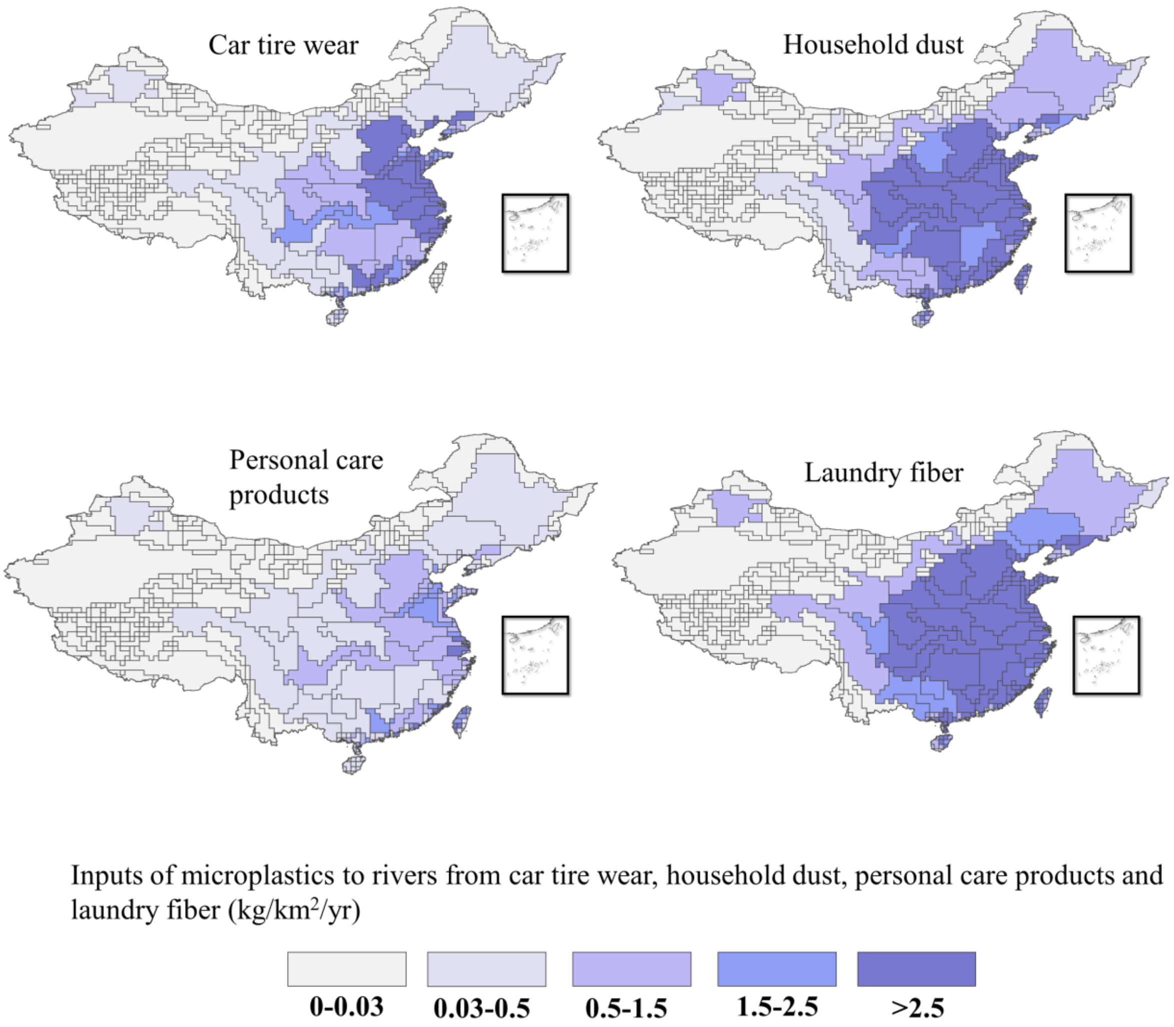

Figure S5. Inputs of microplastics to rivers from car tire wear, household dust, personal care products, and laundry fiber in the year 2015 (kg/km^2/yr). Source: the MARINA-Plastics model (China-1.0), see the model described in the “Materials and Methods” section.

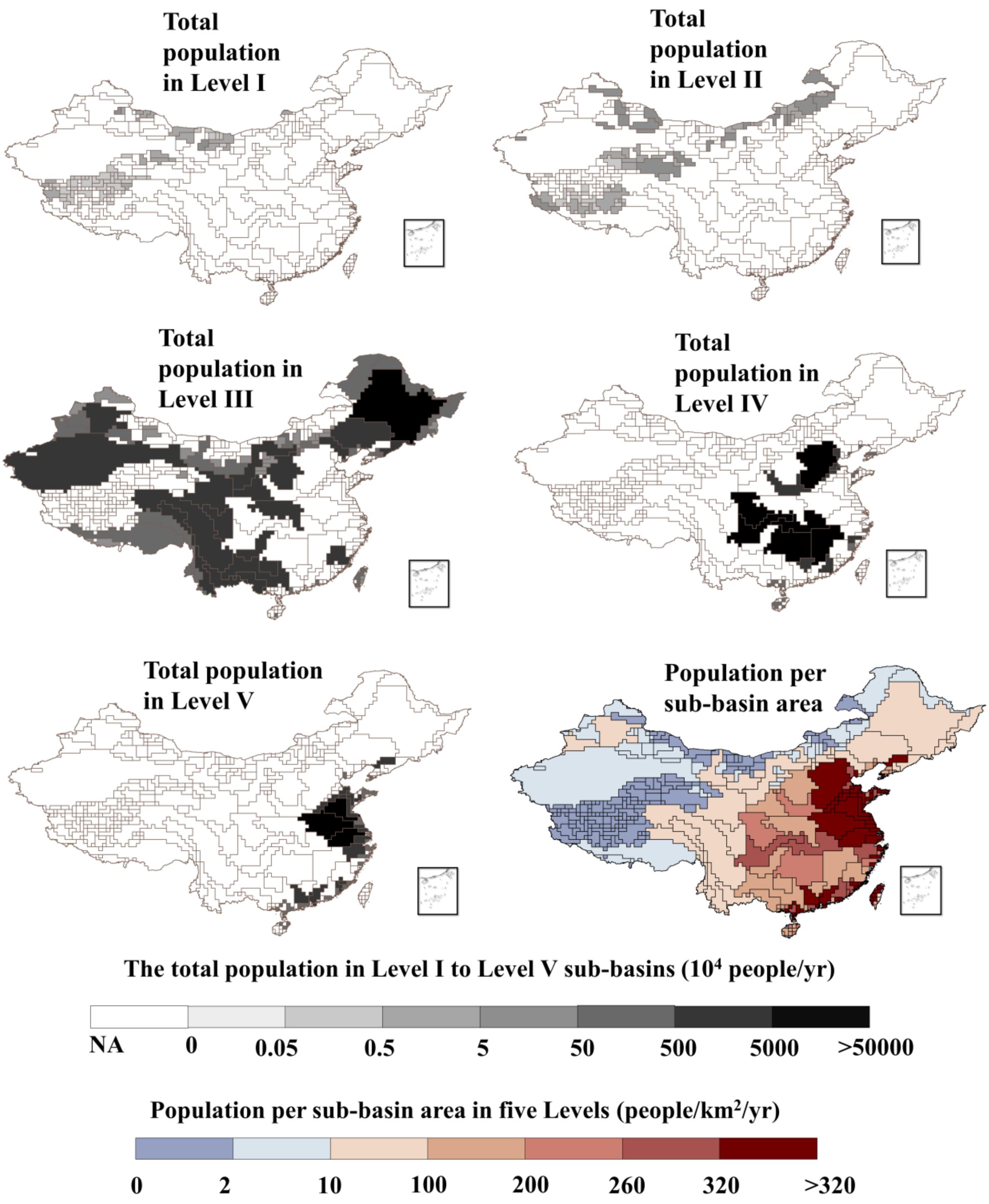

Figure S6. The spatial distribution of population in Chinese sub-basins in the year 2020 (10^4 people/yr and people/km^2/yr). Due to data availability on calculating microplastic inputs to rivers from sewage effluent in rural and urban areas (Details see "Methods" section). We assumed the population in 2015 and 2020 did not change much (only a 2% increase in 2020 [4]). The definition of the five levels is in the main text in Figure 2b. Sub-basins of level IV and V are hotspots area. Source: The MARINA-Plastics model (China-1.0), see the model described in the "Materials and Methods" section.

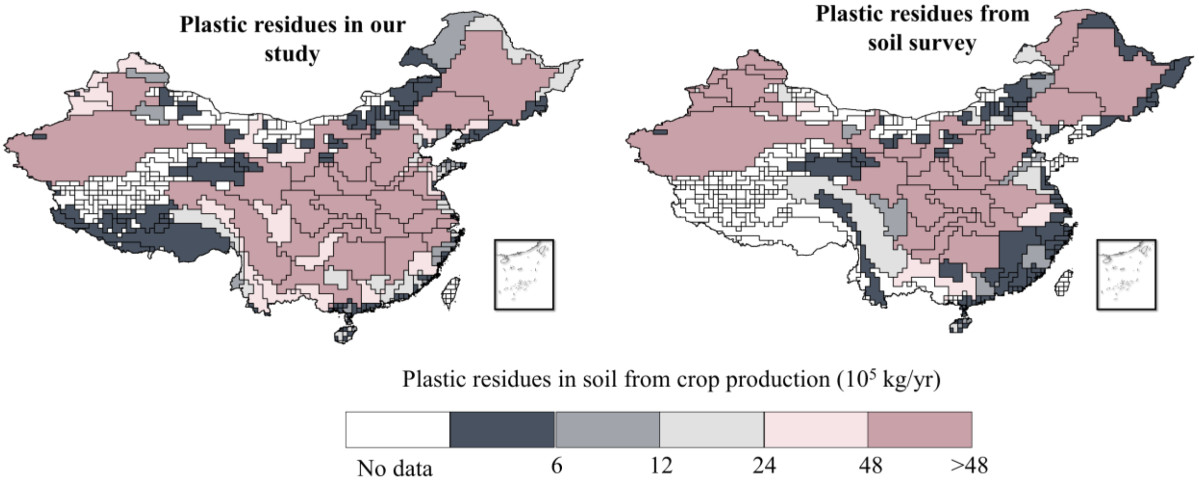

Figure S7. Comparison of our plastic residues with the soil survey data (10^5 kg/yr). Plastics residues are on cropland and result from mulching and greenhouse. We show the results for the sub-basins of our study. Survey data was aggregated to the sub-basins. Source: the MARINA-Plastics model (China-1.0), see the model described in the “Materials and Methods” section; the provincial data on plastic residues in the soil from the survey of the second soil census is from the study of Zhang et al. [5]. We aggerated provincial plastic residue data to the sub-basin scale by using ArcGIS (see Table S4).

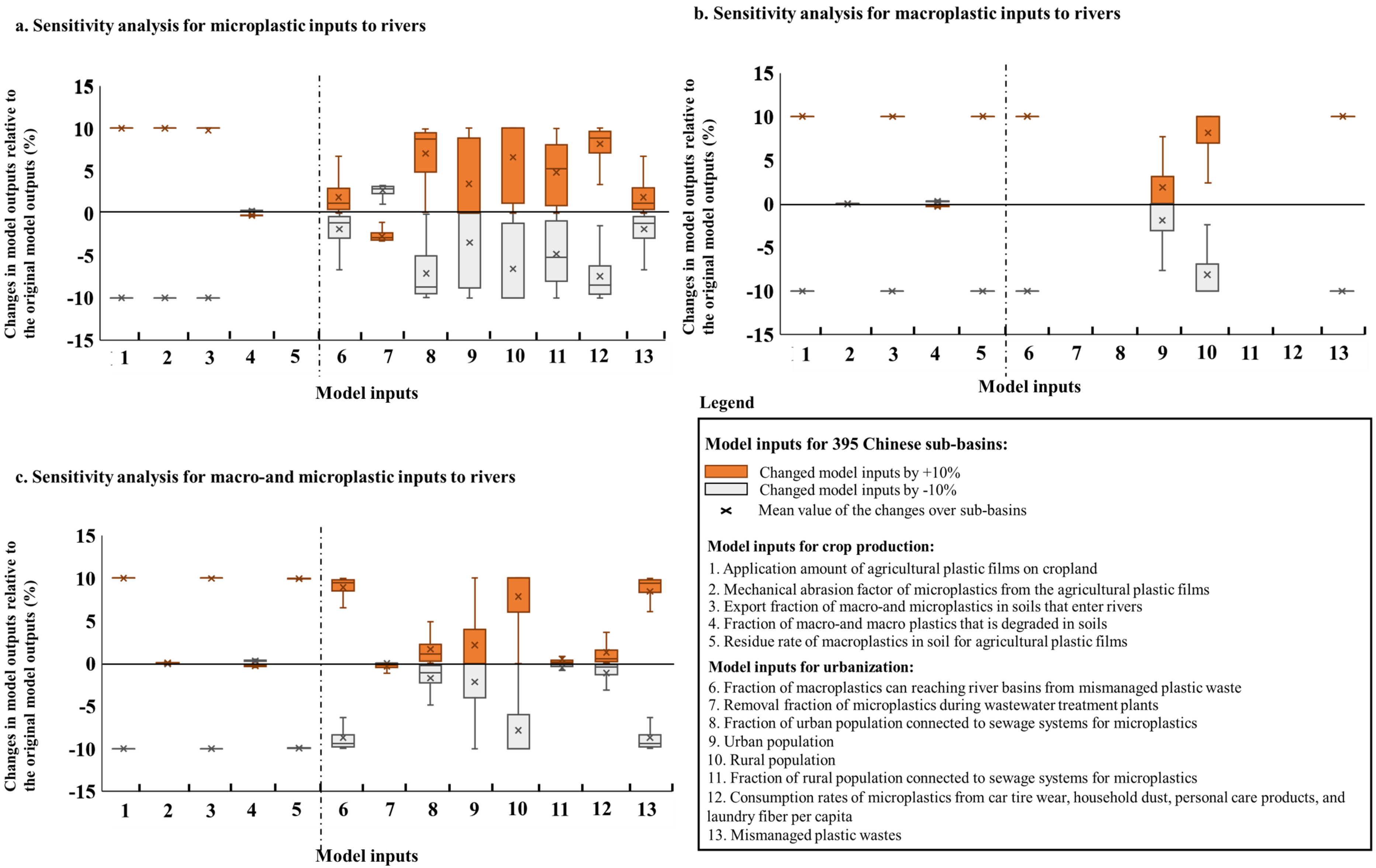
a. Sensitivity analysis for microplastic inputs to rivers
b. Sensitivity analysis for macroplastic inputs to rivers
c. Sensitivity analysis for macro-and microplastic inputs to rivers
Changes in model outputs relative to the original model outputs (%)
15
10
5
0
-5
-10
-15
1 2 3 4 5 6 7 8 9 10 11 12 13
Model inputs
Legend
Model inputs for 395 Chinese sub-basins:
Changed model inputs by +10%
Changed model inputs by -10%
Mean value of the changes over sub-basins
Model inputs for crop production:
1. Application amount of agricultural plastic films on cropland
2. Mechanical abrasion factor of microplastics from the agricultural plastic films
3. Export fraction of macro-and microplastics in soils that enter rivers
4. Fraction of macro-and macro plastics that is degraded in soils
5. Residue rate of macroplastics in soil for agricultural plastic films
Model inputs for urbanization:
6. Fraction of macroplastics can reaching river basins from mismanaged plastic waste
7. Removal fraction of microplastics during wastewater treatment plants
8. Fraction of urban population connected to sewage systems for microplastics
9. Urban population
10. Rural population
11. Fraction of rural population connected to sewage systems for microplastics
12. Consumption rates of microplastics from car tire wear, household dust, personal care products, and laundry fiber per capita
13. Mismanaged plastic wastes

Figure S8. Results of the sensitivity analysis for the MARINA-Plastics (China-1.0) model. This figure shows the changes in the model outputs relative to the original model run (%). Model outputs are annual input of microplastics (a), macroplastics (b), and plastics (c) to rivers of 395 sub-basins. We changed 13 model inputs by ± 10% relative to the original model values. Numbers 1-5 indicate model inputs that are related to plastics from crop production (agricultural plastic films). Numbers 6-13 indicate model inputs that are related to plastics from urbanization-related sources (sewage systems and mismanaged solid waste). The orange color indicates changes in model inputs by +10% relative to the original model run. The grey color indicates changes in model inputs by -10% relative to the original model run. Sources: the MARINA-Plastics (China v1.0) model (the description is in the "Materials and Method" section).

Table S1. An overview of plastics-related policies or action plans in China.

**Policies or action plans**	**Year**	**Description**
Law of the People's Republic of China on the Prevention and Control of Environment Pollution Caused by Solid Wastes	2007	This law concerns domestic waste disposal, clean production, rationally utilizing solid waste, and hazardless treat solid waste [6]
Notice on Restricting the Production and Sale of Plastic Shopping Bags	2008	It is forbidden the production, sell, and use of plastic products (e.g., bags) that have a thickness below 0.025 mm plastic; This document promotes the establishment of payment systems for plastic bags during retail, and the improvement of plastic waste recycling systems [7].
Provisions on the Administration of Prevention and Control of Environmental Pollution by Processing and Utilization of Waste Plastics	2012	This document promotes the improvement of plastic waste recycling, processing, reusing, and treatment of hazardous plastic waste [8].
Environmental protection law of the people's republic of China	2014*	This law concerns solid waste management, waste sorting, recycling, and disposal [9].
Prohibition of Foreign Waste Entering the Country and Promotion of the Implementation Plan for the Reform of the Solid Waste Import Management System	2017	The document highlights that the import of solid waste from foreign countries is forbidden, and strictly administrated the transport and use of solid waste [10].
Agricultural Film Recycling Action Plan	2017	This action plan aims at improving the recycling of agricultural plastic films and reducing their application; 100 demonstration counties are established for plastic film control in Xinjiang province, Inner Mongolia province, and Gansu provinces [11].
Garbage Classification Action	2019	The action highlights garbage classification and recycling. There are four categories for garbage classifications which include dry garbage, wet garbage, recyclable garbage, and hazardous garbage [12].
Soil Pollution Prevention and Control Law of the People's Republic of China	2019	This law specifies that agricultural material producers, sellers, and farmers need to recycle plastic films on time in crop production. The law promotes better supervision of the utilized plastic materials from agricultural activities (e.g., agricultural plastic films, pesticides, fertilizers). The local government is asked to make plans for the construction of urban and rural domestic sewage treatment plants and waste disposal facilities [13].
Administrative measures for agricultural film	2020	These measures request agricultural film producers to produce high-quality agricultural films which should meet the national standard. The government encourages multi-party involvements in agricultural film recycling to reduce the plastic residue on agricultural land [14].
"Opinions on Further Strengthening the Control of Plastic Pollution"	2020	This document aims to reduce plastic product consumption and encourage to exploration of alternative environmentally friendly materials [15].

Measures for reporting the Use and Recycling of Disposable Plastic Products in the Commercial fielding (Trial)	2020	Measures highlight the reductions in using non-degradable plastic products in shops, markets, catering delivery services, the hospitality industry, and various exhibition activities [16].
Action plan for plastic pollution control of the “14th Five-Year Plan”	2021	This plan aims to reduce disposable plastic products and facilitate cleanup programs for plastics in rivers, reservoirs, and lakes. To improve plastic recycling, the recycling rate of agricultural plastic film should reach 85%, achieving zero growth on plastic residue in cropland [17].
Technical specification for pollution control of plastic waste	2022	This specification focuses on pollution control and environmental management requirements for waste plastics production, collection, transportation, storage, pretreatment, recycling, and disposal [18].

***** The first version was in 1979, and the latest version was in 2014

Table S2. Sources of macro-and microplastics in rivers from crop production and urbanization related sources. Sources: MARINA-Plastics (China-1.0) in the "Materials and Methods".

Sources	Point sources	Diffuse sources
Urbanization related sources	Microplastics from Sewage systems	Macroplastics from the Mismanaged solid waste
	-	Microplastics released from macroplastics
Crop production	-	Macroplastics from agricultural plastic films
	-	Microplastics from agricultural plastic films

Table S3. Sources of the data for model inputs. The abbreviations are explained in the main text “Materials and Methods”.

Model inputs	Units	Value	Sources	Related equation in the main text	Code in Supplementary Table S3 for processing methods
**Macro-and microplastics input to rivers from agricultural Plastic films which are applied in crop production**					
APF_j	kg/yr	-	[19]	eq.2	A
MF_j*	unitless	0.001874 or 0.000771	[20]	eq.2	B
$fr_{resdue,j}$	0-1	-	[5]	eq.3	C
$fr_{deg,j}$	0-1	-	Estimated	eq.4-5	D
$FEsr_j$	0-1	-	[21]	eq.4-5	E
**Macro-and microplastics input to rivers from urban and rural sources**					
$WScap_{mic.j}$,	kg/cap/year	0.12 for laundry<br>0.18 or 0.018 for tires<br>0.0071 for personal care products<br>0.08 for household dusts	[22, 23]	eq.7	F
$hw_{mic.sew.j}$	0-1	-	[23]	eq.7	G
Urb_j and Rur_j	People/year	-	[24]	eq.17	H
$fr_{urb.con.j}$ $fr_{rur.con.j}$	0-1	-	[23]	eq.17	-
Pop_j	People/year		[24]	eq.16	I
$WSdif_{mac.j}$,	kg/cap/year	-	[25]	eq.16	J
$F_{leakage,j}$	0-1	-	Estimated	eq.15	K
$t_{res.s.j}$	years	5	[26]	eq.14	-
F_{mac}	0-1	0.03	[26]	eq.9	-
FR_f	0-1	0.95	[26]	eq.10	-
FR_s	0-1	0.05	[26]	eq.11	-
$Area_{land.j}$	km^2	-	[27]	eqs.12-13	L
$Area_{average}$	km^2	1264804	Estimated [23]	eqs.12-13	M

*this parameter is influenced by the solar radiation. Details can be founded in the Table S4.

Table S4. Descriptions of how agricultural model inputs are processed to the sub-basins for the MARINA-Plastics model (China-1.0). The abbreviations are explained in "Methods" of the main manuscript.

CODE (from Table S3)	**MODEL INPUTS**	**DESCRIPTION OF HOW MODEL INPUTS ARE PROCESSED TO SUB-BASINS IN OUR STUDY**	**OUTPUTS**
A	$\boldsymbol{APF_j}$	$\boldsymbol{APF_j}$ is the application amount of agricultural plastic films in sub-basin (j) (kg/yr). This input was available from the Chinese Statistical yearbooks [19]. Agricultural plastic films include plastics on land from mulching and greenhouses. The data on agricultural plastic films was available at the provincial level. We aggregated the provincial data to sub-basins by following five steps below.<br><br>**<u>First</u>,** we prepared a map with the cropland data per province (km^2 per province). This was done by summing the cropland data of 0.5-degree cells over the corresponding provinces. The cropland data of 0.5-degree cells was available from the IMAGE model (Integrated Model to Assess the Global Environment) [28]. According to the information from literature, we assumed that agricultural plastic films can be applied on three types of agricultural land: (1) agricultural land for wetland rice (2); agricultural land for legumes; (3) and agricultural land for upland crops (e.g., Wheat, maize). We used the ArcGIS zonal statistic function to sum agricultural land area of 0.5-degree cells over corresponding Chinese provinces. As a result, we obtained a map with the cropland per province (km^2).<br><br>**<u>Second</u>,** we calculated the application rate of agricultural plastic films per province (kg/km^2 of province/yr). This was done by dividing the total application amount of agricultural plastic films in 2015 (kg/yr per province) by the total cropland (km^2 per province) at the provincial level.<br><br>**<u>Third</u>,** we assigned the provincial application rates (kg/km^2 of province/yr) to the grids of 0.5-degree cells (kg/km^2 of grid/year) using ArcGIS. This implies that we overlapped the map of the delineated provinces with the map of the delineated grids. All grids belonging to the province received the application rates of that province.<br><br>**<u>Fourth</u>,** we multiplied the gridded application rate of agricultural plastic films (kg/km^2 per grid/yr) with the gridded crop land area to obtain the total application of agricultural plastic films per grid (kg/yr).<br><br>**<u>Fifth</u>,** we summed the total application of agricultural plastic films for sub-basins over the corresponding grids to obtain the total application of agricultural plastic films per sub-basin (kg/yr).	Figure S3
B	$\boldsymbol{MF_j}$	$\boldsymbol{MF_j}$ is the mechanical abrasion factor of microplastics from agricultural plastic films in sub-basin (j) (unitless). This factor is based on the study of Ren et al. [20]. They performed experiments and identified the correlations between the relative light transmittance and emission factors of microplastics from agricultural plastic films. They considered the thickness and plastic materials, namely Polyethylene (PE) and polyvinyl chloride (PVC). For our study, we used the results of the experimental work of Ren et al. [20]: we averaged statistically the mechanical	This fraction is used to calculate microplastic entering rivers from crop production (Figure S4)

		abrasion factors of microplastics from PE and PVC plastic films. Ren et al. [20] indicated that higher light transmittance generally leads to a higher mechanical abrasion of microplastics from agricultural plastic films. Thus, in our study, we also considered the influence of solar radiation.<br><br>We used the averaged mechanical abrasion factors of microplastics from PE and PVC plastic films from Ren et al. [20]. These factors are not specific per sub-basin or province. To make them specific, we used the spatial distribution of solar radiation at the sub-basin scale to obtain the mechanical abrasion factors per sub-basin and followed the three steps below.<br><br>**<u>First</u>,** we prepared a raster map with land area data of 0.5-degree cells (km^2). We used the ArcGIS raster calculator function to convert the unit km^2 to m^2 by multiply km^2 with 10^6. Then, we used the zonal statistic function to sum grid areas of 0.5-degree cells over the corresponding Chinese sub-basins. As a result, we obtained a map with the land area per sub-basin (m^2).<br><br>**<u>Second</u>,** we calculated the average solar radiations per sub-basins using the second and third steps. In this step, we identified the spatial distribution of solar radiation in 395 Chinses sub-basins. We took the processed average surface solar radiation data in 12 months of 0.5-degree cells from the GloWPa model [3] (kJ/m^2). We summed the monthly solar radiation data over 12 months at 0.5-degree cells to get annual solar radiation (kJ/m^2). Then, we used the annual solar radiation of 0.5-degree cells (kJ/m^2) and multiply it with land area data of 0.5-degree cells (m^2) to get the solar radiation per 0.5-degree cells (kJ per gird). After that, we summed the solar radiation of 0.5-degree cells over the corresponding Chinese sub-basins. This was done by using ArcGIS zonal statistic function. The output of this step is a map with spatial distribution of the solar radiation in 395 Chinses sub-basins (kJ).<br><br>**<u>Third</u>,** we calculated the solar radiation per land area in each sub-basin (kJ/m^2 land area). This was done by the solar radiation per sub-basin (kJ) (the result of the second step) divided by land area per sub-basin (m^2) (the result of the first step). After that, we assigned the mechanical abrasion factors to sub-basins (unitless) using the information on the solar radiation in sub-basins (kJ/m^2 per sub-basin). The mechanical abrasion factors are based on the study of Ren et al. [20] (we averaged factors over PE and PVC plastic films). The averaged mechanical abrasion factors for both PE and PVC are 0.000771 and 0.001874. According to the knowledge of Ren et al. [20] higher solar radiations generally lead to higher mechanical abrasions of microplastics. Thus, we ranked solar radiations per land area in sub-basin from high to low. We assigned 0.001874 to the sub-basins that have higher solar radiation (198 sub-basins). Then, the rest sub-basins (197) are assigned with a value of 0.000771. As a result we obtained the mechanical abrasion factor of microplastics from agricultural plastic films for each sub-basin.	
C	$\boldsymbol{fr_{resdue,j}}$	$\boldsymbol{fr_{resdue,j}}$ is the residue rate of macroplastics in soils for sub-basin (j) (0-1). Data for the residue rate is based on the study of Zhang et al. [5]. They calculated the amount of plastic residues in the soil after mulching and greenhouse were collected. However, they did not consider the mechanical abrasion of microplastics. Therefore, we correct the values of Zhang et al. [5] for the mechanical abrasion in this study. The values of the residue rate from Zhang et al. [5] were based on field experiments that were scattered across the Chinese provinces. These experiments were conducted in the year 2017. We used this information to calculate provincial plastic residue rate in soils, corrected for the mechanical abrasion and aggregated them to our sub-basins. We assumed that	This fraction is used to calculate microplastic entering rivers from crop production (Figure S4)

		plastic management in agriculture in 2015 was similar to plastic management in 2017. Thus, we used the available information of 2017 [5] to represent the situation of 2015 in our study.<br><br>We calculated the plastic residue rate in the soil for provinces and aggerated them to the sub-basins following four steps below.<br><br>**<u>First</u>,** we prepared the application of agricultural plastic films (mulching and greenhouses) per province (kg/yr) in 2017 using data from the Chinese Statistical yearbook [29].<br><br>**<u>Second</u>,** we collected the plastic residue data from the study of Zhang et al. [5]. Zhang et al. [5] collected the proportion of mulching and greenhouse plastic collected per province (given in %). Then, they calculated the plastic residue from mulching and greenhouse in 2017 per province (kg/yr). This was done as follows: the application amount of greenhouse plastic (kg/yr) × (100%- % of greenhouse plastics that are collected) + the application amount of mulching (kg/yr) × (100%-% of mulching plastics are collected). However, this residue amount is not yet corrected for the mechanical abrasion of microplastics during mulching and greenhouse practices. To obtain the residue amount which is corrected for the mechanical abrasion, we performed the following steps below.<br><br>**<u>Third</u>,** we assigned the mechanical abrasion of microplastics to provinces based on the spatial distribution of the solar radiation (see this step in $\boldsymbol{MF_j}$, but for provinces). Then, we took agricultural plastic films in 2017 (kg/yr) (from the first step) and multiplied it with the mechanical abrasion factor (unitless). In this way, we obtained the amount of microplastics in the soil (kg/yr per province) that has resulted from mechanical abrasion processes in 2017. These values were for provinces. After that, we calculated the amount of macroplastics on land after the mechanical abrasion-related process (see Figure 1). This was done as follows: the application of agricultural plastic films per province in 2017 (kg/yr) (from the first step) minus microplastics from the mechanical abrasion-related process (kg/yr) per province (see the step above). The result of this step resulted in macroplastics on land that is corrected for the mechanical abrasion, but not corrected for plastic collection during crop growth and harvesting. For that we performed the fourth step below.<br><br>**<u>Fourth</u>,** we calculated the residue rate of macroplastics in the soil per province (0-1). This was done by using the plastic residue from mulching and greenhouse in 2017 per province (kg/yr) (from the study of Zhang et al. [5], the result from the second step) and divided it with the amount of macroplastics in the soil per province in 2017 (kg/yr) (we calculated in the third step). We used the area-weighted approach to aggregated the provincial macroplastic residue rate to sub-basins (Figure S2).	
D	$\boldsymbol{fr_{deg,j}}$	$\boldsymbol{fr_{deg,j}}$ is the fraction of macro-and microplastics that are degraded (deg) in the soil in sub-basins (j) (0-1). Plastic degradations could be influenced by biological, physical, and chemical processes (Table S3). We performed a literature review to better understand which factors might be related to those processes [30-33]. For example, degradation rates of plastics associated with biological conditions could be related soil organic matter characteristics [34-49]. Degradation rates associated with physical conditions could be related to the solar radiation intensity [33, 50-53]. And, degradation rates associated with chemical conditions could be related to soil pH [30]. In	Figure S1<br><br>This fraction is used to calculate microplastic entering rivers from

		general, higher organism activities in the soil may promote more plastic degradation. Soil organic matter content may reflect this. Likewise, higher solar radiations generally lead to higher degradation rates of plastics. Acidic conditions could facilitate plastic degradations compared to neutral conditions in which degradations are generally lower. Existing literature shows that plastics can be degraded in the soils ranging from 0.01 to 0.05 (the fraction) depending on biological, physical, and chemical processes (Table S4) [30, 33-50]. We used the information on soil organic matter, solar radiation and pH, and knowledge on the biological, physical and chemical processes from literature to estimate the degradation rates of plastics in the soil. We assumed the same importance for the physical, chemical, and biological process to macro-and microplastics in soil.<br><br>We estimated the fraction of macro-and microplastics that are degraded in the soil (0-1) in our sub-basins following four steps below.<br><br>**First,** we collected the solar radiation (kJ/m^2), pH (unitless) and soil organic matter (g soil organic matter/ kg soil) at a grided scale. Solar radiation data was from the GloWPa model at the resolution of 0.5-degree cells [3]. Soil pH and soil organic matter data were from the Nation Earth System Science Data Center (NESSDC) at the resolution of 90 m × 90 m and 1km × 1km, respectively [1, 2].<br><br>**Second,** we aggerated grided solar radiation (kJ/m^2), pH (unitless) and soil organic matter (g soil organic matter/ kg soil) to sub-basins using ArcGIS. We followed the three steps as described for $\boldsymbol{MF_j}$ (see above) to obtain the solar radiation data per sub-basins. For pH, we had the gridded data. We overlapped the pH data per grid over the sub-basins. We identified the majority of the grids with the same pH values. This implies that the pH value was dominant in the grids of the sub-basins. Therefore, we assigned that dominant pH value to sub-basins. We used the zonal statistic function in ArcGIS to sum soil organic matter of 1 m × 1 m degree cells over the corresponding sub-basins to obtain soil organic matter per sub-basin.<br><br>**Third**, we assigned the degradation rates from the literature to each sub-basin (0-1) based on the solar radiation, pH, and soil organic matter data prepared in the second step (see also details in Table S3). Solar radiation data was used as a proxy to reflect physical processes for plastic degradations. pH was used as a proxy to reflect chemical processes. Soil organic matter was used to reflect biological processes for plastic degradations in the soil. For solar radiation, we ranked solar radiation values for the sub-basins from high to low. Then, we clustered the ranked soil radiation values into five equal intervals (20%, 40%, 60%, and 80%). This implies that the top 20% of sub-basins had the highest soil radiation values. Therefore, we assigned a high degradation rate of 0.05 to those sub-basins. We did the same for the other sub-basins. For the sub-basins belonging to the class from 20% to 40% (based on the rank of the soil radiation data), we assigned the degradation rate of 0.04. For the sub-basins belonging to the class from 40% to 60%, we assigned the degradation rate of 0.03. For the sub-basins belonging to the class from 60% to 80%, we assigned the degradation rate of 0.02. For the sub-basins belonging to the class from 80% to 100%, we assigned the degradation rate of 0.01. We applied a similar approach to assign the degradation rates of plastics using the data for soil organic matters. However, we applied a different approach to assign the degradation rates of plastics using pH data. The values of pH are from 4, 5, 6, 7, to 8 for 395 Chinese sub-basins. According to the knowledge from existing literature (step 1 and Table S3), we assigned the degradation plastic rate of 0.05 (derived from literature, see above) to the sub-basins with pH of 4. We assigned	crop production (Figure S4)

		the degradation plastic rate of 0.04 to the sub-basins with pH of 5. We assigned the degradation rate of 0.03 to the sub-basins with pH of 6. We assigned the degradation rate of 0.01 to the sub-basins with pH of 7. Sub-basins with pH of 8, we assigned the degradation rate of 0.02. As a result, each sub-basin received three degradation rates reflecting physical (based on solar radiation values), chemical (pH values), and biological (soil organic matter) processes for plastics in the soil.<br><br>**Fourth,** we averaged statistically the three fractions of plastic degradation rates in the soil (0-1) per sub-basin (see Figure S1).	
E	$\boldsymbol{FEsr_j}$	$\boldsymbol{FEsr_j}$ is the export fraction of macro-and microplastics in soils that enter rivers via surface runoff (sr) in sub-basin (j) (0-1). The fraction is calculated based on the approach of Zhang et al. [54]. Zhang et al. [54] used the annual observed runoff that was divided by annual precipitation to analyze the effects of the ecological restoration in 16 basins located in the plateau in China from the year 1961 to 2015. In our study, we used a similar approach to calculate the fraction of macro-and microplastics in soils that enters rivers with surface runoff in the 395 Chinese sub-basins. This fraction is applied to the amount of plastics in the soil that is potentially available to be exported by surface runoff to rivers while accounting for degradation. The calculation of $\boldsymbol{FEws_j}$ was done in three steps.<br><br>**First,** we prepared the 30 years of annual precipitation data (mm/yr) (1980-2010) from the WFDEI Meteorological Forcing datasets [21]. We averaged statistically 30 years of annual precipitation of 0.5-degree cells over the sub-basins to obtain the 30 years averaged annual precipitation per sub-basin (mm/yr). This was done by using the ArcGIS zonal statistic function. The output of this step is a map with the average annual precipitation per sub-basins (mm/yr) within 30 years (1980-2010).<br><br>**Second,** we calculated the 30 years of annual runoff per sub-basin (mm/yr). This was done by dividing the 30 years average annual natural river discharge in sub-basins (km^3/yr) by the drainage area (km^2/yr) of these sub-basins. Then, we converted 30 years of average annual runoff from km/yr to mm/yr by km/yr multiply it with 10^6. Natural river discharge and drainage area data per sub-basin were taken from the MARINA-Multi (Global-1.0) model at the sub-basin scale [55].<br><br>**Third,** the export fraction of macro-and microplastics in soils that enter rivers in the sub-basin was calculated by dividing the average annual runoff (mm/yr) (the second step) by the average annual precipitation (mm/yr) for each sub-basin (the first step). To build trust in our export fraction, we collected the runoff and precipitation from the Year Book of China Water Resources in 2011 at the basins scale [56]. We divided the average annual runoff (mm/yr) per basin (defined by the China Statistics Yearbook) with the average annual precipitation (mm/yr) per basin to obtain the export fraction at a basin scale. This export fraction at the basin scale (from the Year Book of China Water Resources) was comparable with our export fraction at the sub-basin scale (our study).	This fraction is used to calculate microplastic entering rivers from crop production (Figure S4)
F	$\boldsymbol{WScap_{mic.j}}$	$\boldsymbol{WSdif_{mic.j}}$ is the consumption rate of microplastics (mic) per capita (cap) in sub-basin (j) (kg/cap/yr). We considered the consumption rate of microplastics including car tyre, laundry fibers, personal care products, and	This fraction is used to calculate

		household dust. The consumption rate of laundry fibers, personal care products, and household dust are constant and derived from Siegfried et al.[22] (See Table S2). The consumption rate of the car tire depends on HDI (Human Developed Index) following the approach of [23].<br>○ If HDI>0.785, $WScap_{tire.j}$ = 0.18<br>○ If HDI<0.785, then $WScap_{tires.j}$ = 0.018<br>Higher HDI reflects a society that is more developed and can afford more cars, implying more microplastic production from car tires. In those sub-basins, the amount of microplastics from car tire is assumed to be higher.<br><br>Four steps are conducted to calculate the $\boldsymbol{WScap_{mic.j}}$ per sub-basin.<br>**<u>First,</u>** we collected the provincial HDI from Chinese Human development report in 2016.<br><br>**<u>Second,</u>** we used the area-weighted approach to aggregate the provincial HDI to sub-basins (Figure S2).<br><br>**<u>Third,</u>** we assigned the consumption rate of microplastic from car tires to sub-basins following the HDI values per sub-basin. For example, sub-basins for which HDI is higher than 0.785 are assigned with the rate of 0.18 kg of microplastics/cap/yr. This is according to the method of Siegfried et al.[22]**.**	microplastic entering rivers from sewage systems (Figure S5)
G	$\boldsymbol{hw_{mic.sew.j}}$	$\boldsymbol{hw_{mi.j}}$ is the removal fraction of microplastics (mic) during sewage treatment (sew) in sub-basin (j) (0-1). This fraction is directly took from the study of Strokal et al. [23], who processed the country data for primary, secondary and tertiary treatment and their removal efficiencies to sub-basins. The approach for calculating the averaged removal fraction for microplastics during treatment process in the study of Strokal et al. [23] is based on the approach of Siegfried et al [22]. Details can be found in the study of Strokal et al. [23].	This fraction is used to calculate microplastic entering rivers from sewage systems (Figure S5)
H, I	$\boldsymbol{Pop_j}$, $\boldsymbol{Urb_j}$ **and** $\boldsymbol{Rur_j}$	$\boldsymbol{Pop_j}$ is the total population in sub-basin (j). $\boldsymbol{Urb_j}$ is for urban population. $\boldsymbol{Rur_j}$ is for rural population. We used the same approach to aggregate $\boldsymbol{Pop_j}$, $\boldsymbol{Urb_j}$ **and** $\boldsymbol{Rur_j}$ to sub-basins:<br><br>**<u>First</u>**, we derived rural and urban populations from the study of Jones and O'Neill [24], which were presented at the grid of 0.5- degree cells.<br><br>**<u>Second</u>**, we aggregated gridded population to sub-basins by using ArcGIS zonal statistic functions by summing the population over the corresponding grids.	Figure S6 for the total population
J	$\boldsymbol{WScap_{mac.j}}$	$\boldsymbol{WScap_{mac.j}}$ is the consumption of macroplastics (mac) per capita in sub-basin (j) (kg/cap/yr). We derived the total amount of mismanaged plastic waste from Lebreton and Andrady [57]. The original data was by provided at the country level in kg/year for 2015. We aggregated the national data to sub-basins by using five steps:	Figure S3

		**First,** we calculated the consumption rate of mismanaged plastic waste (kg/capita/yr) by using the total mismanaged plastic waste (kg/yr) divided by the national total population (people/yr);<br><br>**Second,** we assigned the national production rate of mismanaged plastic waste (kg/capita/yr) to the grids of 0.5-degree cells (kg/km^2 of grid/year) using ArcGIS. All grids receive the same production rate of mismanaged plastic waste.<br><br>**Third,** we used the function of the raster calculator in ArcGIS to calculate the total production of mismanaged plastic waste for the year 2015 (kg/yr) at a gridded scale. This was done by multiplying the production rate of grided mismanaged plastic waste (kg/capita/yr) per capita with the grided population density (people/year, see point H, I row).<br><br>**Fourth**, we summed the total production of mismanaged plastic waste for sub-basins over the corresponding grids to obtain the total mismanaged plastic waste per sub-basin (kg/yr).<br><br>**Fifth,** we used the total mismanaged plastic waste per sub-basin (kg/yr) to total population per sub-basins (people/yr) to get the mismanaged plastic waste per capita for sub-basins (kg/capita/year).	
K	$\boldsymbol{F_{leakage,j}}$	$\boldsymbol{F_{leakage,j}}$ is the fraction of macroplastic that can reach river sub-basins (j). We assigned the leakage rate of 1-5% to sub-basins based on the sub-basin characteristics of soil pH, organic matter and solar radiation. The leakage rate of 1-5% was identified based on the studies of [26, 58]. Details are shown in the aforementioned D row.	This fraction is used to calculate microplastic entering rivers from mismanaged plastic waste (Figure S4)
L	$\boldsymbol{Area_{land.j}}$	$\boldsymbol{Area_{land.j}}$ is the total land area of sub-basin (j) (km^2). We directly took this value from the study of Strokal et al. [23].	This fraction is used to calculate microplastic entering rivers from mismanaged plastic waste (Figure S4)
M	$\boldsymbol{Area_{average}}$	$\boldsymbol{Area_{average}}$ is the average land area of the 50 largest river basins globally. We directly took this value from the study of Strokal et al. [23]	This fraction is used to calculate microplastic entering rivers from mismanaged plastic waste (Figure S4)

Table S5. The fraction of plastic degradations in the soil as influenced by biological, physical, and chemical processes (0-1). This data is based on a literature review.

Processes	Descriptions	Ranges for the fraction of plastic degradations in the soil based on lab experiments	References
**Biological**	Presence of organisms with the capacity to ingest and degrade plastics	<0.01-0.05	[34-49]
**Physical**	Exposure to solar radiation	0.02-0.06	[33, 50-53]
**Chemical**	Exposure to acids or alkalis	0.01-0.7	[30]

Table S6. **Comparisons of our modeled microplastic inputs to rivers in Chinese sub-basins with other studies (kton/yr)**. Source: our modeled results are from the MARINA-Plastics model (China-1.0, see the model described in the "Methods" section).

Year	Region	Value (kton/yr)	Findings	References
2015	China	145	Macro-and microplastics in rivers from agriculture	Our MARINA-Plastics model
2015	China	103	Microplastics in in rivers from point sources	Our MARINA-Plastics model
2015	China	120	Microplastics entering the aquatic environment	[59]
2018	China	120-220	Microplastics input to rivers from agriculture	[60]

Table S7. Comparisons of our modeled microplastics in the soil from crop production with experimental data between 1999 and 2021 (kg/km^2/yr) **(kg/km^2/yr).** Crop production includes plastics from mulching and greenhouses. The location of provinces and sub-basins are presented in Figure S7.

Provinces	Experimental studies			Our study	
	Year	Value	References	Year	Value
Jiangsu	2019	0.14-0.24	[61, 62]	2015	0.4-3
Shandong	2017-2020	0.05-0.82	[63-67]	2015	0.3-2.4
Gansu	2011-2016	0.05-3.7	[67-70]	2015	0.06-1
Ningxia	1999-2020	0.13-0.3	[71, 72]	2015	0.005-1
Inner Mongolia	2015-2020	0.37-1.4	[63, 66, 69, 73-75]	2015	0.0001-0.4
Xinjiang	2013-2020	0.19-3.8	[67, 69, 76-87]	2015	7×10^{-6}-0.4
Shaanxi	2014-2016	0.3-2.09	[67, 88, 89]	2015	0.01-1
Shanxi	2017	1.8	[90]	2015	0.1-0.4
Henan	2018	0.1-0.7	[67, 91, 92]	2015	0.3-2.6
Qinghai	2017	0.21-0.84	[93, 94]	2015	0.001-0.3
Hebei	2008-2016	0.053-1	[67, 92, 95]	2015	0.06-1
Yunnan	2013-2015	0.17-0.4	[96, 97]	2015	0.05-0.3
Liaoning	2019	0.2-1.6	[98]	2015	0.06-1.6
Heilongjiang	2005-2011	0.03-0.04	[87, 99, 100]	2015	0.01-0.08
Si Chuan	2011-2017	0.06-0.16	[101, 102]	2015	0.08-0.3
Hubei	2017	0.7-1.4	[67]	2015	0.2-0.5

References

1. NESSDC, China High Resolution National Soil Information Grid Basic Attribute Dataset_90m Soil pH (2010-2018). In Center, S. S.; Center, N. E. S. S. D.; China, N. S. T. I. o., Eds. http://soil.geodata.cn, 2022 a.
2. NESSDC, Map of soil organic matter content on a 1-kilometer grid in China (1990). In SubCenter, S.; Center, N. E. S. S. D.; China, N. S. T. I. o., Eds. http://soil.geodata.cn, 2022 b.
3. Vermeulen, L. C.; Hengel, M.; Kroeze, C.; Medema, G.; Spanier, J. E.; Vliet, M. T. H. v.; Hofstra, N., Cryptosporidium concentrations in rivers worldwide. *Water Research* **2019,** *149*, 202-214.
4. NBSC, China Statistic Yearbook. In China, N. B. o. S. o., Ed. China Statistic Press: Beijing, 2021.
5. Zhang, Q.-Q.; Ma, Z.-R.; Cai, Y.-Y.; Li, H.-R.; Ying, G.-G., Agricultural Plastic Pollution in China: Generation of Plastic Debris and Emission of Phthalic Acid Esters from Agricultural Films. *Environmental Science & Technology* **2021,** *55*, 12459−12470.
6. MOA, Law of the People's Republic of China on the Prevention and Control of Environment Pollution Caused by Solid Wastes. In China, M. o. C. P. s. R. o., Ed. http://english.mofcom.gov.cn/aarticle/policyrelease/internationalpolicy/200703/20070304471567.html, 2007.
7. CSC, Notice on Restricting the Production and Sale of Plastic Shopping Bags. In China, G. O. o. t. S. C. o. t. P. s. R. o., Ed. https://www.fao.org/faolex/results/details/en/c/LEX-FAOC142871, 2008.
8. MEE, Provisions on the Administration of Prevention and Control of Environmental Pollution by Processing and Utilization of Waste Plastics. In China, M. o. E. a. E. o. t. p. s. r. o., Ed. 2012.
9. NPC, Environmental protection law of the people's republic of China. In Committee, N. P. s. C. N. S., Ed. http://www.gov.cn/zhengce/2014-04/25/content_2666434.htm, 2014.
10. CSC, Prohibition of Foreign Waste Entering the Country and Promotion of the Implementation Plan for the Reform of the Solid Waste Import Management System. In China, G. O. o. t. S. C. o. t. P. s. R. o., Ed. https://leap.unep.org/countries/cn/national-legislation/implementation-plan-prohibiting-entry-foreign-garbage-and, 2017.
11. MOA, Agricultural Film Recycling Action Plan. In China, M. o. A. a. R. A. o. t. P. s. R. o., Ed. http://www.moa.gov.cn/nybgb/2017/dlq/201712/t20171231_6133712.htm, 2017.
12. Tong, Y.-q.; Liu, J.-f.; Liu, S.-z., China is implementing "Garbage Classification" action. *Environmental Pollution* **2020,** *259*, 113707.
13. MEE, Law of the People's Republic of China on Prevention and Control of Soil Contanmination. In China, M. o. E. a. E. T. P. s. R. o., Ed. https://english.mee.gov.cn/Resources/laws/environmental_laws/202011/t20201113_807786.shtml, 2019.
14. MOA, Administrative measures for agricultural film. In China, M. o. a. a. R. A. o. t. P. s. R. o., Ed. https://leap.unep.org/countries/cn/national-legislation/administrative-measures-agricultural-film, 2020.
15. NDCR, Opinions on Further Strengthening the Control of Plastic Pollution. In Commission, N. D. a. R., Ed. https://www.ndrc.gov.cn/xxgk/zcfb/tz/202001/t20200119_1219275.html?code=&state=123, 2020.
16. MOC, Measures for reporting the Use and Recycling of Disposable Plastic Products in the Commercial fielding (Trial). In China, M. o. C. o. t. P. s. R. o., Ed. http://www.mofcom.gov.cn/article/zwgk/zcfb/202011/20201103019141.shtml, 2020.
17. NDRC, Action plan for plastic pollution control of the "14th Five-Year Plan". In Comission, N. D. a. R., Ed. http://www.gov.cn/zhengce/zhengceku/2021-09/16/content_5637606.htm, 2021.
18. MEE, Technical specification for pollution control of plastic waste. In China, M. o. E. a. E. o. P. s. R. o., Ed. https://www.mee.gov.cn/ywgz/fgbz/bz/bzwb/gthw/qtxgbz/202206/t20220607_984652.shtml, 2022.
19. NBSC, China Rural Stastical Yearbook In Bureau, C. S., Ed. Beijing: China Statistics Press, 2016.
20. Ren, S.-Y.; Sun, Q.; Ni, H.-G.; Wang, J., A minimalist approach to quantify emission factor of microplastic by mechanical abrasion. *Chemosphere* **2020,** *245*, 125630.
21. Weedon, G. P.; Balsamo, G.; Bellouin, N.; Gomes, S.; Best, M. J.; Viterbo, P., The WFDEI meteorological forcing data set: WATCH Forcing Data methodology applied to ERA-Interim reanalysis data. *Water Resource Research* **2014,** *50*, 7505–7514.
22. Siegfried, M.; Koelmans, A. A.; Besseling, E.; Kroeze, C., Export of microplastics from land to sea. A modelling approach. *Water Research* **2017,** *127*, 249-257.
23. Strokal, M.; Bai, Z.; Franssen, W.; Nynke, H.; Koelmans, A. A.; Ludwig, F.; Ma, L.; van Puijenbroek, P.; Spanier, J. E.; Vermeulen, L. C.; van Vliet, M. T. H.; van Wijnen, J.; Kroeze, C., Urbanization: an increasing source of multiple pollutants to rivers in the 21st century. *Urban Sustainability* **2021,** *1*, 24.
24. Jones, B.; O'Neill, B. C., Spatially explicit global population scenarios consistent with the Shared Socioeconomic Pathways. *Environmental Research Letters* **2016,** *11*, 084003.
25. Lebreton, L.; Andrady, A., Future scenarios of global plastic waste generation and disposal. *Palgrave Communications* **2019,** *5*, (1), 1-11.
26. van Wijnen, J.; Ragas, A. M. J.; Kroeze, C., Modelling global river export of microplastics to the marine environment: Sources and future trends. *Science of The Total Environment* **2019,** *673*, 392-401.
27. Bodirsky, B. L.; Popp, A.; Weindl, I.; Dietrich, J. P.; Rolinski, S.; Scheiffele, L.; Schmitz, C.; Lotze-Campen, H., N₂O emissions from the global agricultural nitrogen cycle – current state and future scenarios. *Biogeosciences* **2012,** *9*, (10), 4169-4197.
28. Beusen, A.; Bouwman, A.; Beek, L. V.; Mogoll´on, J.; Middelburg, J., Global riverine N and P transport to ocean increased during the twentieth century despite increased retention along the aquatic continuum. *Biogeosci. Discus* **2015,** *12*, 20123–20148.
29. NBSC, *China statistical yearbook (In Chinese).* China Statistic Press: China: National Bureau of Statistics of China, 2017.
30. Ariza-Tarazona, M. C.; Villarreal-Chiu, J. F.; Hernández-López, J. M.; Rosa, J. R. D. l.; Barbieri, V.; Siligardi, C.; Cedillo-González, E. I., Microplastic pollution reduction by a carbon and nitrogen-doped TiO2:

Effect of pH and temperature in the photocatalytic degradation process. *Journal of Hazardous Materials* **2020,** *395*, 122632.

31. Llorente-García, B. E.; Hernández-López, J. M.; Zaldívar-Cadena, A. A.; Siligardi, C.; Cedillo-González, E. I., First Insights into Photocatalytic Degradation of HDPE and LDPE Microplastics by a Mesoporous N–TiO2 Coating: Effect of Size and Shape of Microplastics. *Coatings* **2020,** *10*, (7), 658.
32. Pham, T.-H.; Do, H.-T.; Thi, L.-A. P.; Singh, P.; Raizada, P.; Wu, J. C.-S.; Nguyen, V.-H., Global challenges in microplastics: From fundamental understanding to advanced degradations toward sustainable strategies. *Chemosphere* **2021,** *267*, 129275.
33. Sharma, S.; Basu, S.; Shetti, N. P.; Nadagouda, M. N.; Aminabhavi, T. M., Microplastics in the environment: Occurrence, perils, and eradication. *Chemical Engineering Journal* **2021,** *408*, 127317.
34. Dey, A. S.; Bose, H.; Mohapatra, B.; Sar, P., Biodegradation of Unpretreated Low-Density Polyethylene (LDPE) by Stenotrophomonas sp. and Achromobacter sp., Isolated From Waste Dumpsite and Drilling Fluid. *Front Microbiol* **2020,** *16*, (11), 603210.
35. Ya, H.; Jiang, B.; Xing, Y.; Zhang, T.; Lv, M.; XinWang, Recent advances on ecological effects of microplastics on soil environment. *Science of The Total Environment* **2021,** *798*, 149338.
36. Samanta, S.; Datta, D.; Halder, G., Biodegradation efficacy of soil inherent novel sp. Bacillus tropicus (MK318648) onto low density polyethylene matrix. *J. Polym. Res* **2020,** *27*, 324.
37. Steinmetz, Z.; Wollmann, C.; Schaefer, M.; Buchmann, C.; David, J.; Tröger, J.; Muñoz, K.; Frör, O.; Schaumann, G. E., Plastic mulching in agriculture. Trading short-term agronomic benefits for long-term soil degradation? *Science of the Total Environment* **2016,** *550*, 690-705.
38. Sangale, M. K.; Shahnawaz, M.; Ade, A. B., Potential of fungi isolated from the dumping sites mangrove rhizosphere soil to degrade polythene. *Scientific Reports* **2019,** *9*, 5390.
39. Zhang, X.; Li, Y.; Ouyang, D.; Lei, J.; Tan, Q.; Xie, L.; Li, Z.; Liu, T.; Xiao, Y.; Farooq, T. H.; Wu, X.; Chen, L.; Yan, W., Systematical review of interactions between microplastics and microorganisms in the soil environment. *Journal of Hazardous Materials* **2021** *418*, 126288.
40. Hou, L.; Xi, J.; Liu, J.; Wang, P.; Xu, T.; Liu, T.; Qu, W.; Lin, Y., Biodegradability of polyethylene mulching film by two Pseudomonas bacteria and their potential degradation mechanism. *Chemosphere* **2022,** *286*, 131758.
41. Deepika, S.; Jaya, M. R., Biodegradation of low density polyethylene by micro-organisms from garbage soil. *Journal of Experimental Biology and Agricultural Sciences* **2015,** *3*, (1), 15-21.
42. Roy, P. K.; Hakkarainen, M.; Varma, I. K.; Albertsson, A.-C., Degradable Polyethylene: Fantasy or Reality. *Environmental Science & Technology* **2011,** *45*, 4217–4227.
43. Huang, D.; Xu, Y.; Lei, F.; Yu, X.; Ouyang, Z.; Chen, Y.; Jia, H.; Guo, X., Degradation of polyethylene plastic in soil and effects on microbial community composition. *Journal of Hazardous Materials* **2021,** *416*, 126173.
44. Abraham, J.; Ghosh, E.; Mukherjee, P.; Gajendiran, A., Microbial Degradation of Low Density Polyethylene. *Environmental Progress & Sustainable Energy* **2017,** *36*, 147-154.
45. Albertsson, A. C.; Andersson, S. O.; Karlsson, S., The mechanism of biodegradation of polyethylene. *Polym. Degrad. Stab* **1987,** *18*, 73–87.
46. Bläsing, M.; Amelung, W., Plastics in soil: Analytical methods and possible sources. *Science of the total environment* **2018,** *612*, 422-435.
47. Albertsson, A. C., The shape of the biodegradation curve for low and high density polyethenes in prolonged series of experiments. *European Polymer Journal* **1980,** *16*, (7), 623-630.
48. Vimala, P. P.; Mathew, L., Biodegradation of polyethylene using Bacillus subtilis. *Proced. Technol.* **2016,** *24*, 232–239.
49. Montazer, Z.; Habibi, M. B. N.; Levin, D. B., Microbial degradation of lowdensity polyethylene and synthesis of polyhydroxyalkanoate polymers. *Can. J. Microbiol* **2019,** *65*, (3), 224–234.
50. Auta, H. S.; Emenike, C. U.; Jayanthi, B.; Fauziah, S. H., Growth kinetics biodeterioration of polypropylene microplastics by Bacillus sp. and Rhodococcus sp. isolated from mangrove sediment. *Marine Pollution Bulletin* **2018,** *127*, 15–21.
51. Zhang, J.; Gao, D.; Li, Q.; Zhao, Y.; Li, L. i.; Lin, H.; Bi, Q.; Zhao, Y., Biodegradation of polyethylene microplastic particles by the fungus Aspergillus flavus from the guts of wax moth Galleria mellonella,. *Science of The Total Environment* **2020,** *704*, 135931.
52. Park, S. Y.; Kim, C. G., Biodegradation of micro-polyethylene particles by bacterial colonization of a mixed microbial consortium isolated from a landfill site. *Chemosphere* **2019,** *222*, 527–533.
53. Chen, Z.; Zhao, W.; Xing, R.; Xie, S.; Yang, X.; Cui, P.; Lü, J.; Liao, H.; Yu, Z.; Wang, S.; Zhou, S., Enhanced in situ biodegradation of microplastics in sewage sludge using hyperthermophilic composting technology. *384* **2020,** *Journal of Hazardous Material*, 121271.
54. Zheng, H.; Miao, C.; Zhang, G.; Li, X.; Wang, S.; Wu, J.; Gou, J., Is the runoff coefficient increasing or decreasing after ecological restoration on China's Loess Plateau? *International Soil and Water Conservation Research* **2021,** *9*, 333-343.
55. Li, Y.; Wang, M.; Chen, X.; Cui, S.; Hofstra, N.; Kroeze, C.; Ma, L.; Xu, W.; Zhang, Q.; Zhang, F.; Strokal, M., Multi-pollutant assessment of river pollution from livestock production worldwide. *Water Research* **2022,** *209*, 117906.
56. NBSC, Statistic Year Book of China Water Resources. In China Statistic Press: China: National Bureau of Statistics of China, 2011.
57. Lebreton, L.; Andrady, A., Future scenarios of global plastic waste generation and disposal. *Palgrave Communications* **2019,** *5*, (1), 6.
58. Sundt, P.; Schulze, P.-E.; Syversen, F. *Sources of microplastic pollution to the marine environment*; 2014.
59. Wang, T.; Li, B.; Zou, X.; Wang, Y.; Li, Y.; Xu, Y.; Mao, L.; Zhang, C.; Yu, W., Emission of primary microplastics in mainland China: invisible but not negligible. *Water Research* **2019,** *162*, 214–224.

60. Ren, S.-Y.; Kong, S.-F.; Ni, H.-G., Contribution of mulch film to microplastics in agricultural soil and surface water in China☆. *Environmental Pollution* **2021,** *291*, 118227.
61. Yu, B.; Han, Y.-F.; Ding, J.-P.; Wu, J.-F.; Yu, D.; Pan, G.-Y.; Wang, F., Current Situation and Development Countermeasures of Recycling and Utilization of Agricultural Film Residual Film in Rudong County (in Chinese). *South China Agriculture* **2019,** *13*, (17), 170-171.
62. Mao, B.-C.; Liao, K.-Z.; X-L, M.; Gu, Q.-H., Investigation and analysis of soil film residue in Donghai County. *Bulletin of Agricultural Science and Technology* **2019,** *08*, 213-215.
63. Zhang, F.-s.; Lu, X.; Yao, Y.; Feng, Z.-G., Present situation of the residue pollution of farmland mulch in Tongliao area and its control measures (in Chinese). *Journal of Northern Agriculture* **2020,** *48*, (5), 125-129.
64. Wang, Z.-g., Current Situation and Control Countermeasures of Residual Film Pollution of Farmland in Shandong Province (in Chinese). *Agricultural Engineering Technology* **2019,** *5*, 5.
65. Li, Y.-c.; Li, H.-p.; Wang, Y.-x.; Sun, Y.-p.; Wang, K.-r.; Yang, Q.-x., The current situation and countermeasures of residual film pollution of farmland soil in Qingdao City (in Chinese). *Journal of Agricultural Resources and Environment* **2017,** *34*, (3), 8.
66. Zuo, M.-h.; Liu, B.; Yao, Z.-k.; Zhang, S.-y.; Yu, X.-x.; Li, Y.-z., Investigation and control measures of residual film in farmland in Kailu County (in Chinese). *Modern Agriculture* **2020**, (8), 3.
67. Xu, Y.-m.; Fang, S.-j.; Ma, X.; Zhu, Q., Prevention and Control Strategy for the Pollution of Agricultural Plastic Film (in Chinese). *Chinese Engineering Science* **2018,** *20*, (5), 7.
68. Du, Z.-y.; Sun, D.-x.; Yang, R.; Su, Y.-z., Distribution characteristics and influencing factors of plastic film residues in Zhangye oasis farmland (in Chinese). *Journal of Agricultural Environmental Science* **2020,** *39*, (12), 2789-2797.
69. Huang, Y.; Liu, Q.; Jia, W.; Yan, C.; Wang, J., Agricultural plastic mulching as a source of microplastics in the terrestrial environment. *Environment Pollution* **2020,** *260*, 114096.
70. Ma, Y.; Yang, D.-H., Investigation on Pollution Caused by Mulching Plastic Film in Gansu Province and the Countermeasures (in Chinese). *Journal of Ecology and Rural Environment* **2015,** *31*, (4), 478-483.
71. Yuan, Q.-y.; Yu, J.-L., Countermeasures and suggestions of agricultural plastic film residue pollution in central and Southern Ningxia (in Chinese). *Ningxia Journal of Agriculture and Forestry Science and Technology* **2020,** *61*, (08), 42-44.
72. Yang, S.-m.; Dong, X.-l.; Chen, F., Research on the current situation and control measures of agricultural film pollution in Ningxia. *Ningxia Agriculture and Forestry Science and Technology* **1999**, (6), 43-46.
73. Bai, Y.-l.; Li, X.-l.; Zhang, S.; Bao, L.-h.; Liu, L.-p.; Lin, L.-l.; Zheng, H.-c., Study on the current situation of plastic film residue pollution and the countermeasures for the recovery and utility of residual film in Inner Mongolia (in Chinese). *China soil and fertilizer* **2015**, (6), 139-145.
74. Zhou, X.-c., Current Situation and Suggestions of Plastic Film Residues in Ningcheng County (in Chinese). *Primary Agricultural Technology Extension 2017, Vol., No.* **2017,** *5*, (12), 111-112.
75. Zhang, B.; Wang, Z.; Jin, S., Current situation and prospect of agricultural film pollution treatment in China. *World environment* 2019, pp 22-25.
76. Zhao, Y.; Chen, X.-g.; Wen, H.; Zhang, X.; Niu, Q.; Kang, J.-m., Research status and the prospect of control technology for residual plastic film pollution in farmland (in Chinese). *Journal of Agricultural Machinery* **2017,** *48*, (6), 1-12.
77. Cai, F.-l.; Luo, G.-h.; Wang, C.-y., Status Quo and Control Measures of Residual Film Pollution of Farmland in Changji City in Chinese. *Rural Science and Technology* **2016,** *8*, 2.
78. Li, Y.-z.; Chen, H.-y.; Wang, X.-y.; Ma, L., Countermeasures for pollution control of farmland residual film in Changji City (in Chinese). *Xinjiang Agricultural Science and Technology* **2017**, (2), 2.
79. Tian, M.-m., Current Situation and Development Suggestion of Residual Film Pollution Control of Farmland in Changji Prefecture (in Chinese). *Modern Agriculture Technology* **2017**, (12), 2.
80. Du, Y.-c., Research on the current situation and countermeasures of residual pollution of farmland plastic film in Yuepuhu County, Xinjiang. (in Chinese). *Agriculture and Technology* **2019,** *39*, (9), 2.
81. Hu, C.; Wang, X.-f.; Chen, X.; Tang, X.-y.; Zhao, Y.; Yan, C.-r., Current situation and prevention and control strategies of residual film pollution in Xinjiang farmland (in Chinese). *Chinese Journal of Agricultural Engineering* **2019,** *35*, (24), 12.
82. Hu, C., Present situation and prevention and control scheme of plastic film residue pollution in typical areas of Xinjiang. *Agricultural Engineering Technology* **2019,** *39*, (36), 39-45.
83. Bao, Z.; Lei, L.; Tang, Q.-x., Pathway optimization of control route on the soil mulching film pollution in Xinjiang (in Chinese). *Xinjiang Agricultural Mechanization* **2019**, (6), 5.
84. Wang, X.-j.; Cao, S.-l.; Wang, M.; Yin, Y.-k., Research on the current situation, harm and prevention measures of plastic film residues in farmland (in Chinese). *Annual Conference of Chinese Society for Environmental Sciences* **2013**, 5023-5028.
85. Liu, E.-K.; He, W.-Q.; Yan, C.-R., 'White revolution' to 'white pollution'— agricultural plastic film mulch in China. *Environmental Research Letter* **2014,** *9*, 091001.
86. Niu, R. K.; Wang, X. F.; Hu, C.; Hou, S. L.; Lu, B.; Li, J. B., Analysis of the Current Situations of Plastic Films Ｒesidue Pollution of Cotton Field in Xinjiang Aksu Area. *Xinjiang Agricultural Sciences* **2016,** *53*, (02), 283-288.
87. Yan, C.-r.; He, W.-q.; Turne, N. C.; Liu, E.-k.; Liu, Q.; Liu, S., Plastic-film mulch in Chinese agriculture: importance and problems. *World Agriculture* **2014,** *4*, 5.
88. Yang, Y.-y.; Liu, M.-l.; Liu, T.; Liu, W.-t.; Yang, G.-l.; Yang, Z.-s., Study on the Residue Characteristics of Spring Maize Plastic Film in Weibei Dry Plateau (in Chinese). *Shaanxi Agricultural Science* **2016,** *62*, (1), 4-6.
89. Zhang, M.-l.; Hao, Y.-x.; Yang, D.-y.; Zhao, Q.-r.; Wang, Y., Current Situation and Control Countermeasures of Plastic Film Residues in Weinan City (in Chinese). *Agricultural Science and Technology Communications* **2014**, (8), 171-172.

90. Bai, X.; Zhou, H.-p.; Xie, W.-y., Investigation on Utilization Status of Agricultural Film and Residual Membrane Pollution in Typical Areas of Shanxi Province (in Chinese). *Chinese Agricultural Science Bulletin* **2017,** *33*, (36), 99-104.
91. Pei, X.-l. The Present Situation of Agricultural Film Pollution in Henan Province and the Countermeasures for Its Control. Master thesis, Henan agriculture university, 2018.
92. Zhang, D.; Hu, W.-l.; Liu, H.-b.; Du, L.-f.; Xu, Y.; Cheng, Z.-h.; Sun, S.-y.; Wang, H.-y., Characteristics of residual mulching film and residual coefficient of typical crops in North China (in Chinese). *Transactions of the Chinese Society of Agricultural Engineering Technology* **2016,** *32*, (3), 1－5.
93. Li, W.-Y., Current situation and suggestions of farmland residual film recovery in Ping'an District. *Qinghai Agro-Technology Extension* **2017**, (04), 54-55.
94. Wang, S., The current situation and countermeasures of farmland film residue recovery in Huangzhong County, Qinghai Province (in Chinese). *Qinghai Agricultural Technology Promotion* **2017**, (1), 31.
95. Ma, H.; Mei, X.-r.; Yan, C.-r.; He, W.-q.; Li, K., The Residue of Mulching Plastic Film of Cotton Field in North China. *Journal of Agro-Environment Scienc* **2008,** *27*, (2), 570- 573.
96. Zhang, L.; Yang, J.-h.; Han, Y.-l.; Tang, L.-j.; Xu, W.; Yan, S.; Yang, S.-m., Investigation on the Use and Residue of Plastic Film for Main Film-covered Crops in Dehong Prefecture (in Chinese). *China Tropical Agriculture* **2015,** *4*, 35-37.
97. Kang, P.-d.; Hu, Q.; Lu, Y.; Lei, B.-k.; He, G.-q.; Wang, C., Study on plastic film residues in typical corn growing areas in Lijiang Yunnan. *Hunan Agricultural Science* **2013**, (2), 3.
98. Zhao, Q.-w., Current Situation Analysis and Suggestion of Agricultural Plastic Mulch Residue Pollution in Liaoning Province. *Horticulture & Seed* **2019,** *4*, 51-53.
99. Xu, G.; Du, X.-m.; Cao, Y.; Wang, U.-h.; Xu, D.; Lu, G.-L.; Li, F.-s., Residue Levels and Morphology of Agricultural Plastic Film in Representative Areas of China (in Chinese). *Journal of Agro-Environment Scienc* **2005**.
100. Du, X.-m.; Xu, G.; Xu, D.; Zhao, T.; Li, F., Mulch film residue contamination in typical areas of north China and countermeasures. *Transactions of the Chinese Society of Agricultural Engineering* **2005,** *21*, (13), 225-227.
101. Zhou, X.-g.; Rao, Z.-s.; Yang, Y.; Yi, M.; Huang, H.-l.; Wang, G.-h., Analysis on Membrane Residue and Influence Factors of Yibin Tobacco Area in Sichuan (in Chinese). *Anhui Agricultural Sciences* **2018,** *46*, (19), 70-71.
102. Huang, J.-j.; Pang, L.-y.; Luo, C.; Lin, C.-w.; Zeng, Z.-l.; Hu, B.; Wang, L., Residues and influencing factors of plastic film in Panxi area, Sichuan (In Chinese). *Southwest Agricultural Journal* **2012,** *25*, (6), 4.
